# Learnable Diffusion Framework for Mouse V1 Neural Decoding

**DOI:** 10.1002/advs.202520220

**Published:** 2026-03-05

**Authors:** Kaiwen Deng, Peter S. Schwendeman, Yuanfang Guan

**Affiliations:** ^1^ Department of Computational Medicine and Bioinformatics University of Michigan Ann Arbor Michigan USA; ^2^ Department of Electrical Engineering and Computer Science University of Michigan Ann Arbor Michigan USA; ^3^ Department of Internal Medicine University of Michigan Ann Arbor Michigan USA

**Keywords:** diffusion model, mouse, neural decoding, primary visual cortex

## Abstract

Decoding visual stimuli from neural signals is an essential step toward understanding how sensory information is represented in the brain. While most existing approaches reconstruct visual stimuli from human functional magnetic resonance imaging (fMRI), utilizing calcium imaging in mice opens the door to single‐neuron‐level insights into non‐primate visual systems with distinct spectral sensitivities. Here, we present Sensorium‐Viz, a diffusion‐based framework specifically designed for decoding activity in the mouse primary visual cortex. The model is among the first to reliably reconstruct complex, high‐resolution images from previously unseen single‐neuron responses. At its core, Sensorium‐Viz introduces two key advances for neuron‐to‐image decoding: a synthetic‐response augmentation strategy that improves reconstruction performance by more than 30% while enabling cross‐mouse generalization through fine‐tuning, and an architectural design that integrates a Diffusion Transformer (DiT) with a spatial neuron‐embedding module, thereby achieving up to a 10.65% performance gain over leading fMRI‐based reconstruction methods across pixel‐ and content‐level benchmarks. Analysis of the neural responses and corresponding reconstructions reveals that neurons sensitive to low‐level visual features form the primary basis of V1's representation of external stimuli. These findings establish Sensorium‐Viz as a biologically grounded and technically robust tool for vision decoding.

## Graphical abstract

We introduce Sensorium‐Viz, a diffusion‐based framework for reconstructing high‐fidelity visual stimuli from mouse primary visual cortex activity. By integrating a novel spatial embedding module with a Diffusion Transformer (DiT) and a synthetic‐response augmentation strategy, our model outperforms state‐of‐the‐art fMRI‐based baselines, enabling robust cross‐subject generalization and validating biologically grounded feature selectivity in neural decoding.

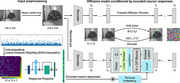

## 1 Introduction

Reconstructing visual stimuli from brain activity has long been a focus of neuroscience and machine learning research, offering valuable insights into how sensory information is processed in the brain and how the brain understands the external world [1, 2, 3]. Recent advancements in neuroimaging and deep learning have enabled the more accurate mapping of brain signals to visual stimuli, thereby expanding the potential for reconstructing complex, high‐quality images from neural data.

Most prior research on visual reconstruction has utilized functional magnetic resonance imaging (fMRI) data from human brains to regenerate colored visual stimuli. fMRI enables non‐invasive observation of population‐level brain activity by tracking blood oxygen levels, revealing correlations between neural responses and visual information [4, 5, 6]. These approaches have primarily focused on retrieving latent representations from fMRI signals and using them together with generative adversarial networks (GANs) to guide image reconstruction [7, 8, 9]. However, the most recent developments in generative AI have further advanced this field, with diffusion models standing out for their exceptional ability to generate semantically rich reconstructions from human brain data [10, 11].

Despite the success of fMRI‐based reconstruction in humans, much less is known about how visual experiences can be reconstructed in other species and with alternative recording modalities.Studying such systems is crucial for validating the generalizability of neural decoding frameworks across different species and recording modalities. In particular, mice have long served as model organisms for non‐primate visual systems [12, 13], and advances in two‐photon calcium imaging with craniotomy now allow researchers to record activity from individual neurons, providing a level of detail not accessible with population‐level measures [14]. Leveraging these capabilities, a pioneering study by Yoshida and Ohki (2020) demonstrated that images could be reconstructed from mouse calcium signals using a cell‐selection linear model on Gabor filter–based features [15]. While this investigation demonstrated the feasibility of neuron‐to‐image decoding, it relied on contrast‐enhanced stimuli that were downsampled to 32 × 32 pixels for reconstruction, along with handcrafted Gabor kernels, which are known to capture mostly low‐level edges and textures [16]. Other studies explored non‐linear models and convolutional neural networks (CNNs), but these efforts were similarly constrained by low‐resolution stimuli and achieved only moderate performances [17, 18].

At the same time, attempts to directly train state‐of‐the‐art human fMRI reconstruction models, such as MinD‐Vis, on mice V1 single‐neuron responses similarly fail to produce semantically meaningful images or accurate pixel‐level alignment [10] (Figure S1A,B). Together, these limitations emphasize the need for models specifically designed for non‐human visual systems and raise several compelling questions: How can advanced AI architectures be adapted to calcium–imaging–based decoding for more complex visual stimuli? How can the limited data available per subject be leveraged efficiently to meet the large‐sample requirements of AI models? And can such valid generative models be established by demonstrating their alignment with the known biological properties of V1 visual coding?

Motivated by these questions, we introduce Sensorium‐Viz, an image reconstruction framework tailored for calcium imaging data from the mouse primary visual cortex. Sensorium‐Viz combines a lightweight spatial embedding module for neuron responses with a Diffusion Transformer (DiT) model, which generates images conditioned on these embeddings. We demonstrate two key advances. First, we describe a synthetic response augmentation strategy that improves reconstruction quality and enables efficient cross‐subject generalization. Second, we show that our architecture outperforms state‐of‐the‐art fMRI‐based approaches under equivalent training and evaluation conditions. Beyond performance, we validate that the model's reconstructions are primarily driven by specific feature‐selective neurons rather than global population activity, consistent with the functional organization of V1. Together, these results establish Sensorium‐Viz as a robust tool for high‐fidelity visual decoding in animal models, offering new opportunities for probing the neural basis of complex visual experiences.

## 2 Results

### 2.1 Sensorium‐Viz Reconstructs Stimuli via DiT With Spatially Embedded Neuron Responses

**FIGURE 1:**
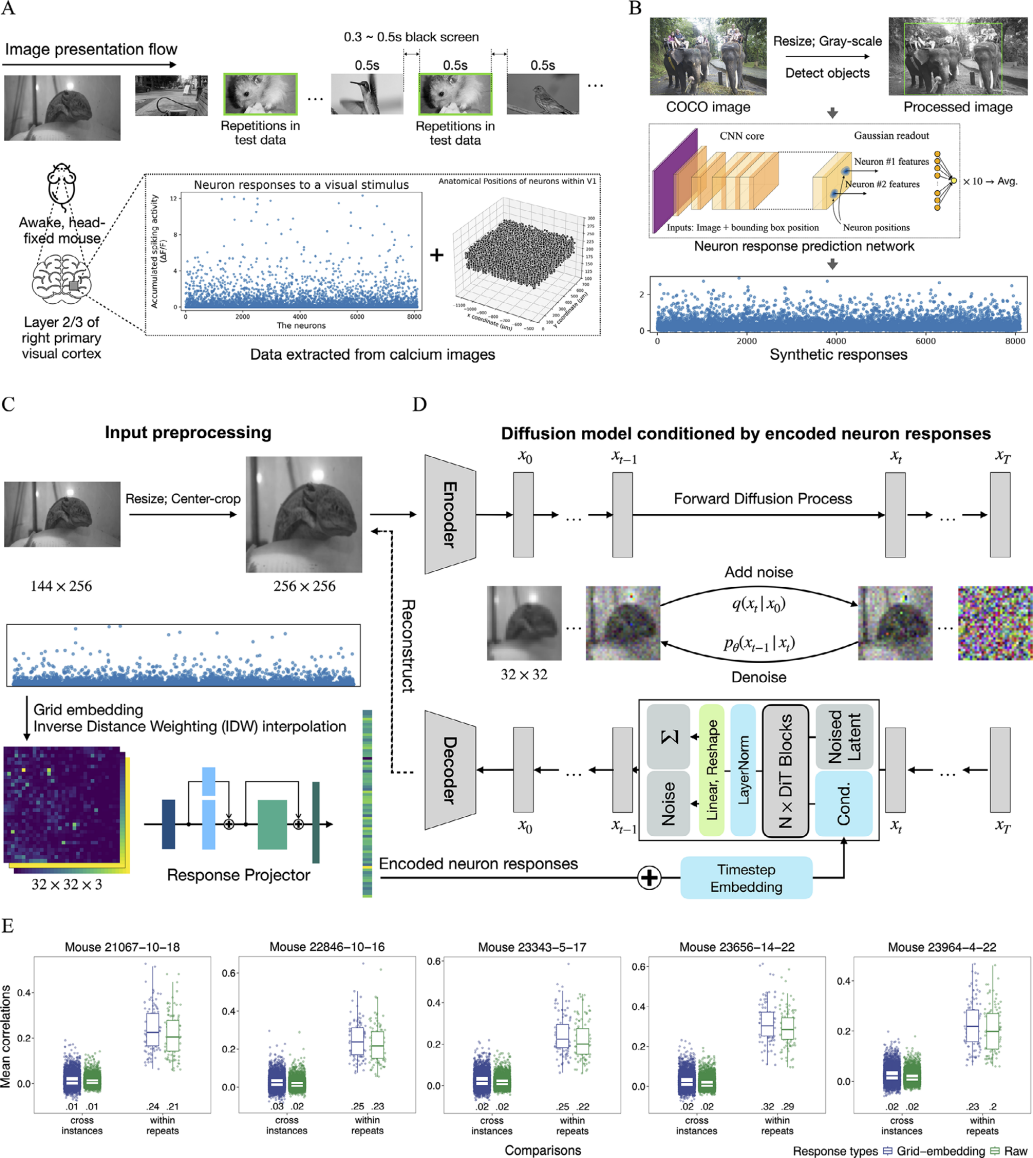
Utilize the Diffusion Transformer (DiT) to reconstruct the visual stimuli conditioned by the neuron responses. (A) describes how the images were presented to the mice in the datasets and the data types used to train the models. The images were shown to awake, head‐fixed mice in a flow with 0.5 s presentation times, followed by 0.3–0.5 s of black screens. There were 100 instances used as the test data in the original datasets, which were repeated 10 times and mixed with the other images in the flow (the image with the green border in this figure is an example). The neuron responses to a specific image, along with their anatomical positions, were extracted from V1 of the mouse brains. (B) shows the pipeline for generating synthetic responses from COCO image sets. The processed images and the object positions in the images, as well as the neuron coordinates of the mice, were given to our pre‐trained neuron signal prediction models to generate the synthetic responses specifically for images and mice. There were 10 pre‐trained models, and the final output was their average. (C,D) draw the complete pipeline of our method. (C) indicates our preprocessing on the image and neuron responses, including resizing the input images and embedding and encoding the neuron response to fit the input shapes of DiT. (D) briefly describes the overall structure of the diffusion model. (E) presents the results of signal correlations comparing the repetitions within the same instances and across different instances. The blue points and boxes are for the responses after spatial embeddings. The green ones are the raw responses. Each point represents the average correlation among the repetitions.

Here, we describe a novel reconstruction model for calcium image data, which we later use to investigate how signals from the primary visual cortex in mouse brains represent and process visual stimuli. To train the model, we used training data obtained from the Sensorium Challenge, which was designed for neural encoding tasks (i.e., predicting the corresponding neural signals from given visual stimuli). This dataset consists of neuronal recordings from five awake, head‐fixed mice presented with grayscale stimuli derived from ImageNet images [19]. We focused on these five subjects because they had fully accessible test sets with repeated trials; the remaining two mice from the original challenge were excluded as they served as held‐out test subjects without publicly available response data. These neuronal responses were recorded from layers 2 and 3 (L2/3) of the right primary visual cortex (V1). The spiking activity of neurons was captured via two‐photon calcium imaging and quantified as relative fluorescence changes. Neuronal responses were accumulated between 50 and 550 ms after stimulus onset using a boxcar window. This specific window was selected to account for the approximately 50 ms transmission latency from the retina to V1 and to fully capture the integrated calcium dynamics corresponding to the 500 ms stimulus duration [20]. Each neuron's response to a given stimulus was represented by a single value. The number of recorded neurons varied across mice (Table 1). Each instance in the training data presented only once and there is no overlap between the training and testing instances. Additionally, the anatomical coordinates of each neuron relative to the pial surface were also recorded (Figure 1A).

**TABLE 1:** Experimental information of the mouse in the Sensorium Challenge datasets.

Mouse ID	Neuron #	Total trials #	Test trials #	Test instance #
21067	8372	5994	998	100
22846	7344	5997	999	100
23343	7334	5951	989	100
23656	8107	5966	993	100
23964	8098	5983	994	100

Sensorium‐Viz employs a Diffusion Transformer (DiT) to model the relationship between visual inputs and neural responses (Figure 1D). DiT utilizes an enhanced denoising function through transformer‐based blocks, similar to those seen in Vision Transformers (ViTs), and encodes external information as conditions using Adaptive Layer Norm (adaLN) Zero blocks (Figure 1D; Figure S2B) [21]. This improved architecture yielded better image generation quality for grayscale images in our preliminary experiments (Figure S1C,D). Models were trained separately for each mouse on its trials, excluding the test ones (Table 1). As with other diffusion models, training occurred in two stages: forward diffusion, where input stimuli are projected into a low‐dimensional latent space and progressively perturbed with Gaussian noise; and reverse denoising, where the denoising function is trained to iteratively remove noise and reconstruct the original input, guided by the neural responses.

Sensorium‐Viz receives the image and neuron response data during training and only requires the latter for back‐inferencing the input images. Images are resized and center‐cropped to a shape of 256 × 256 to fit the model's requirements (Figure 1C). Center cropping minimizes information loss by preserving the image regions most relevant to neural activity [22]. To encode the neuron responses, we account for the noisy nature of neuronal activity and its spatial dependence within the brain. In our previous study, we found that grid‐based embedding methods have shown strong ability in summarizing neuron signals while preserving their spatial properties [24]. Thus, we utilized a similar strategy that embedded the responses into 32 × 32 grids using an Inverse Distance Weighting (IDW) algorithm, which uses interpolated 2D neuron positions as XY coordinates, normalized to the range of −1 to 1. The resulting spatially structured response maps are further processed by a grid response projector network, which encodes them as conditions to guide the DiT model's reconstructions (Figure 1C; Figure S2A).

To validate that the spatial embedding preserves the informational structure of sparse neuronal activity, we performed a representational similarity analysis comparing signal consistency across repeated presentations of the same test stimulus (‘within‐stimulus’) to comparisons between different stimuli (‘cross‐stimulus’). As expected for reliable V1 coding, raw neural responses exhibited a clear separation between signal and noise, with an average correlation of 0.2304 (±0.09) for within‐stimulus repeats compared to 0.0149 (±0.02) for cross‐stimulus instances. Importantly, the spatially embedded response maps maintained this discriminative structure, showing within‐stimulus correlations of 0.2551 (±0.10) and cross‐stimulus correlations of 0.0224 (±0.03) (Figure 1E). This preservation of signal discriminability is further illustrated in correlation heatmaps (Figures S3 and S4), where both raw and embedded responses display distinct high‐correlation diagonal blocks against a low‐correlation background. The similarity between raw and embedded representations confirms that the Inverse Distance Weighting (IDW)–based spatial embedding preserves the representational geometry of V1 responses rather than altering their biological or statistical properties.

### 2.2 Synthetic Responses and Stimulus Repetition Improve the Reconstruction Qualities

**FIGURE 2:**
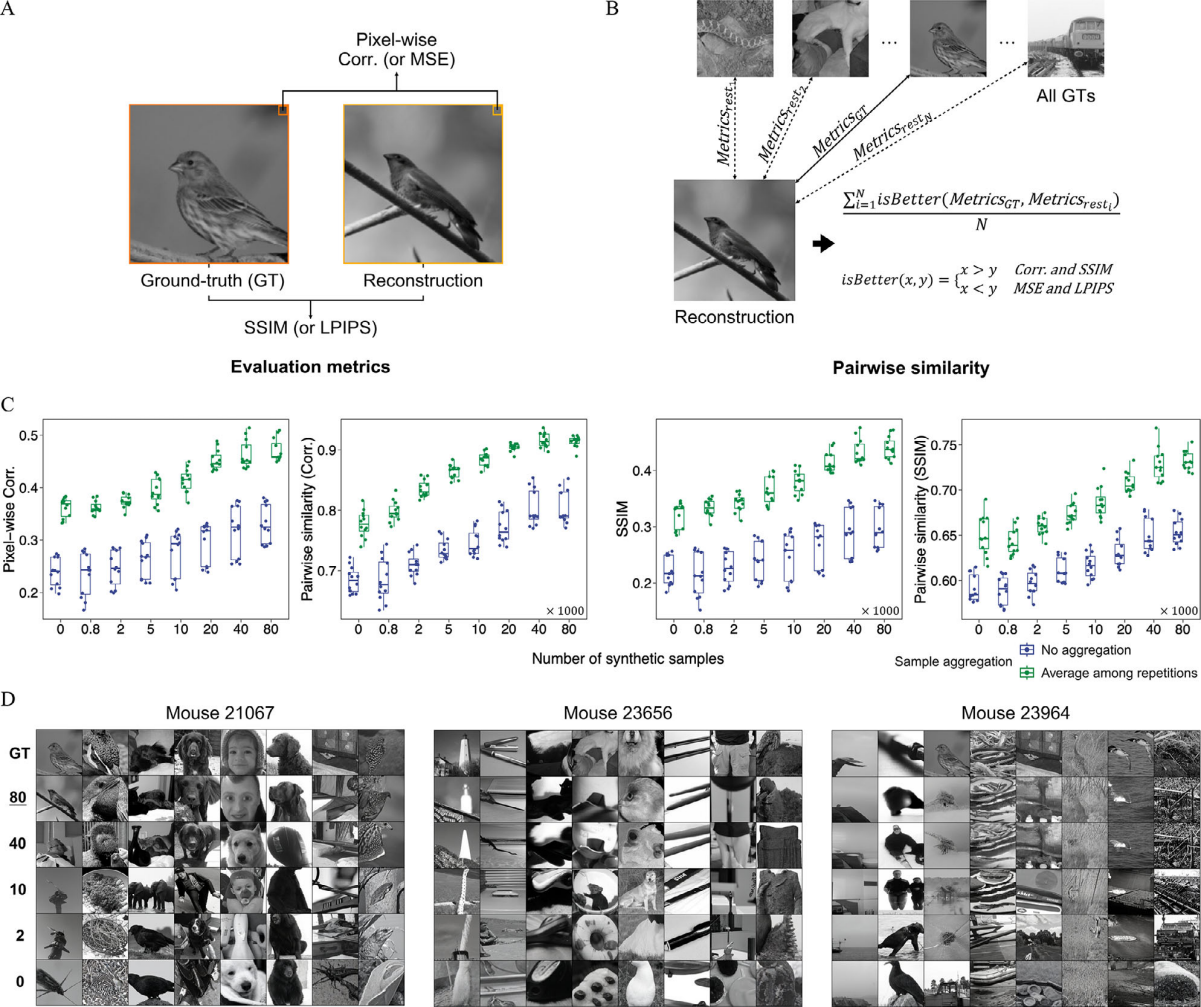
Evaluations of the reconstruction qualities. (A) Overview of the evaluation metrics. SSIM and LPIPS are calculated for the entire image, while MSE and correlations are computed pixel by pixel. (B) Illustration of the pipeline used to compute pairwise similarity for a given metric. The process involves generating scores between the reconstruction and all other ground truths, expecting the metric score for the true ground truth to be the highest. (C) Correlation scores (“Corr.”) and SSIM plots, along with their pairwise similarities, for models trained with different amounts of synthetic data. Results are shown for reconstructions generated from averaged neuronal responses (the green boxes and dots) or from single‐trial, unaveraged responses (the blue boxes and dots). The numbers on the x‐axis represent the real synthetic sample numbers divided by 1000. (D) Sample reconstructions randomly selected from the high‐LPIPS‐score results from three mice, compared with their corresponding ground truths (first row). Reconstructions are shown for various numbers of synthetic samples (listed on the left, consistent with the x‐axis labels in panel (C). All reconstructed images are from the first generation. Full reconstruction results of these three mice can be found in Figures S9,S11, and S13.

We first assessed its reconstruction qualities using test data, which included grayscale images with mostly 10 repetitions of 100 unique instances (Table 1). Five models were trained separately for each of the five mice and evaluated using two metrics (Figure 2A): Pearson's correlation among pixels (pixel‐wise correlation) and the structural similarity index (SSIM), as well as their pairwise similarities, following the reports in previous works (Figure 2B) [8, 23]. These metrics quantify the qualities at the pixel level and of the overall image content. Pixel‐level mean squared error (MSE) and Learned Perceptual Image Patch Similarity (LPIPS) were also reported as additional evaluation metrics [24]. Additionally, since the diffusion model generates images from random noise, we performed four reconstructions per target image. We reported the standard deviations of these metrics to evaluate the stability of the inference.

The task of reconstructing images from neural activity poses significant challenges due to two core limitations of the data. First, neuronal responses are highly noisy and sparsely activated: only a small subset of neurons responds reliably to a given stimulus, while the rest exhibit seemingly random fluctuations. Second, our dataset is relatively small, making it hard for our models to learn generalizable patterns. To mitigate these issues, we apply two key strategies during training and inference to enhance the reconstructions. First, during inference, we reduced trial‐to‐trial variability by averaging the neuronal responses across the 10 repetitions of each test image. For each mouse and each test instance, the merged response vector was obtained by computing the mean response of every recorded neuron among their signals to the repetitions (Figure 2C). Given the low response correlation within instances (Figure 1E), this approach stabilizes the representation of each stimulus. Second, inspired by the training strategy of DALL‐E 3 [25], we augment our dataset with additional training images paired with synthetic responses. To perform this augmentation, we collected 82 784 additional images from the COCO dataset and generated corresponding synthetic neural responses using our previously trained neuron signal prediction model (Figure 1B) [26]. It consisted of a shared module for extracting features from images and the separated Gaussian readout modules that mapped the image features to neuronal responses for each mouse. The training datasets also consist of the trials in Table 1, excluding the test trials.

Applying these strategies resulted in significant improvements in reconstruction performance. After merging the repetitions, the median pixel‐wise correlation and SSIM scores increased by 52.05% and 53.52% on average, respectively. Pairwise similarities also improved by 15.91% and 11.22% (Figure 2C). MSE and LPIPS, along with their pairwise similarities, also showed significant improvements (Figure S6). Incorporating 80 000 additional synthetic samples further enhanced reconstruction quality, leading to 30.43% and 38.20% higher median pixel‐wise correlation and SSIM scores, as well as 17.91% and 12.96% improvements in pairwise similarities (Figure 2C). Visual inspection of the reconstructions confirmed these quantitative results. Models trained with more synthetic samples produced images that more closely resembled the ground truth (Figure 2D). Similar improvements were also observed for MSE and LPIPS (Figure S6).

Among the five mice, the final metric scores of the best‐performing model were 0.4581 for pixel‐wise correlation (min: 0.4367 – max: 0.5092, average std. among the four reconstructions for one image: 0.0069), 0.4235 for SSIM (0.4034 – 0.4722, std.: 0.0060), 0.9191 for pairwise similarity in pixel‐wise correlation (0.8897 – 0.9339, std.: 0.0059), and 0.7309 for pairwise similarity in SSIM (0.7154 – 0.7532, std.: 0.0058) (Figure 2B; Figures S9–S13).

### 2.3 Sensorium‐Viz Outperforms the Previous SOTA fMRI Method

Due to the limited availability of open‐source approaches for calcium imaging‐based reconstructions in mice, we evaluated our model by adapting MinD‐Vis—a state‐of‐the‐art model originally designed for human fMRI data—comparing its performance on data from three mice in our dataset [10]. MinD‐Vis consists of an fMRI embedding module (SC‐MDM) and a conditioned latent diffusion module (DC‐LDM). Considering the differences in data types, we first adjusted the settings of the SC‐MDM module, including the generation of non‐negative outputs and the incorporation of neuron position information. We embedded the neural responses of the mice using the settings that achieved their best performance, with an average correlation between recovered and original signals of 0.7228 (Figure S5). Then, we generated images using the same training and inference strategies as our method, including both synthetic responses and repetition merging.

**FIGURE 3:**
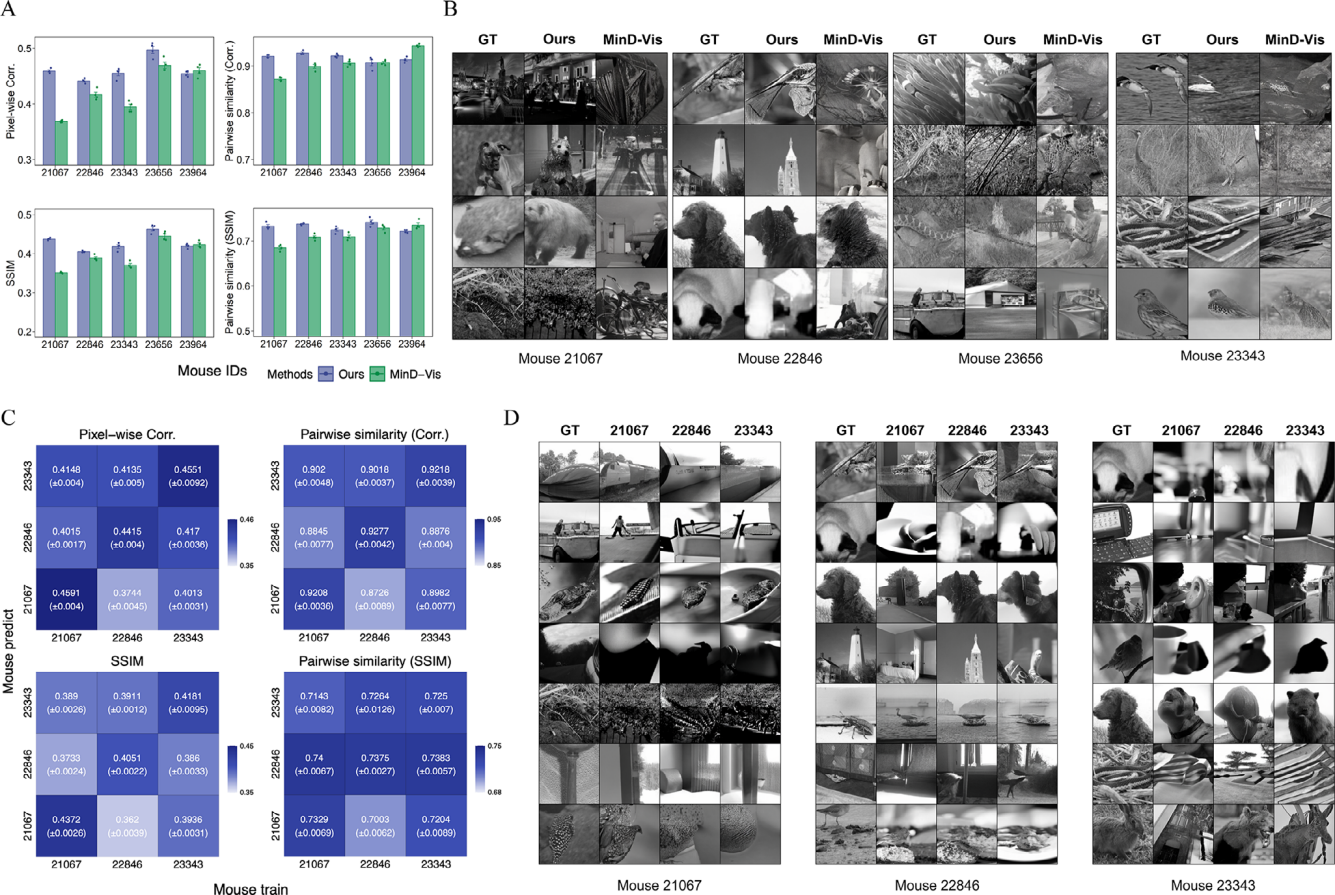
Evaluations of the reconstructions from different methods and cross‐mice inferences. (A) Correlation and SSIM scores, along with their pairwise similarities, are used to compare the performance of our method and MinD‐Vis. Bars represent the median scores from four generations, while scatter points indicate individual scores for each generation. (B) Visual comparison of ground truths and images reconstructed using our method and MinD‐Vis. Our method demonstrates superior reconstruction accuracy at both pixel and semantic levels. (C) Reconstruction performance of transfer‐learning models. The *x*‐axis (“Mouse train”) specifies the mouse datasets (both real and synthetic) used for training the base models, while the *y*‐axis (“Mouse predict”) indicates the synthetic data used for fine‐tuning for each mouse. (D) Decoded images comparing models trained on the same mouse and fine‐tuned on data from other mice. Titles above the images indicate the mouse providing the base model, while titles below indicate the mouse used for fine‐tuning and prediction.

As a result, while performing on‐par with the baseline on mouse 23964, in a general view, Sensorium‐Viz significantly outperformed MinD‐Vis, requiring 11.36x less training time and achieving an average 5.57% higher performance across all major metrics (except MSE) and pairwise similarities. Median correlation and SSIM scores for Sensorium‐Viz were 0.4581 (mean ± s.d.: 0.4615 ± 0.020) and 0.4236 (0.4286 ± 0.021), respectively, compared to 0.4140 (0.4218 ± 0.040) and 0.3862 (0.3955 ± 0.036) for MinD‐Vis (one‐sided paired Wilcoxon test p‐values = 0.006 and 0.0104, respectively). Similarly, median pairwise similarities were 0.9191 (correlation, 0.9181 ± 0.009) and 0.7308 (SSIM, 0.7317 ± 0.009), while MinD‐Vis achieved 0.9037 (0.9056 ± 0.024) and 0.7126 (0.7133 ± 0.024), respectively (one‐sided paired Wilcoxon test *p*‐values = 0.0413 and 0.0076, respectively; Figure 3A; Figure S7). Furthermore, our model produced reconstructions with higher visual fidelity, better preserving fine image details (Figure 3B; Figures S9–S11). These results demonstrate that Sensorium‐Viz more effectively captures the distinctive features of calcium imaging data from the mouse visual cortex compared to the fMRI‐based model, underscoring the viability of our model as a tool for investigating cortical activity in future studies.

### 2.4 Fine‐Tuning with Synthetic Data Enables Efficient Cross‐Mouse Inferences

Due to individual differences among subjects, directly applying a model trained on one mouse to another without adaptation may lead to suboptimal reconstructions. However, training a subject‐specific decoder often demands substantial additional neuronal recordings for each new subject, which is both labor‐intensive and raises ethical considerations. To explore whether synthetic responses could help alleviate this burden, we investigated a transfer strategy in which a model trained on one mouse's data (the “source”) was adapted to another (the “target”) using only synthetic responses. These synthetic responses were generated from encoding models previously developed for the target mouse, which themselves had been trained on a limited amount of real data from that subject.

In this setup, we first trained a base model using both real and synthetic data from a given source mouse, incorporating 80 000 synthetic image–response pairs alongside the approximately 5900 real image–response pairs available per mouse (Table 1). We then fine‐tuned the base model using only synthetic responses generated for a different target mouse, again leveraging 80 000 synthetic samples for adaptation. Finally, the fine‐tuned model was used to reconstruct test images for the target mouse. We conducted this analysis on a subset of three mice (21067, 22846, and 23343) to manage the substantial computational resources required for the all‐to‐all training matrix.

The results show that cross‐mouse fine‐tuning yields reconstruction performance approaching that of models trained entirely on the target mouse's data (Figure 3C; Figure S8). Both correlation and SSIM remain relatively high when transferred across mice, with median scores of 0.4037 (mean ± s.d.: 0.4037 ± 0.015) and 0.3873 (0.3825 ± 0.011), respectively. The median pairwise similarities were 0.9018 (0.8911 ± 0.012) and 0.7263 (0.7233 ± 0.016), respectively.

Notably, these transfer‐learning results are quantitatively comparable to, and in some metrics surpass, the performance of the MinD‐Vis baseline trained directly on the target subject data. On this same subset of mice, MinD‐Vis achieved median scores of 0.3930 (0.3933 ± 0.022) for correlation and 0.3670 (0.3699 ± 0.017) for SSIM, with pairwise similarities of 0.8992 (0.8923 ± 0.017) and 0.7045 (0.7008 ± 0.013). Statistical comparisons confirmed that our cross‐mouse inference performed on par with MinD‐Vis for correlation metrics (*p*‐value = 0.0521 for correlation; p‐value = 0.6844 for pairwise correlation similarity) and significantly outperformed it for SSIM metrics (*p*‐value = 0.0091 for SSIM; p‐value = 0.0004 for pairwise SSIM similarity; one‐sided paired Wilcoxon tests).

Visual inspection of the reconstructed images further confirmed these findings. Cross‐mouse outputs showed similar pixel distribution patterns to both within‐mouse reconstructions and the original ground‐truth stimuli, indicating that key visual features were preserved despite differences in the underlying neuronal recordings (Figure 3D). Taken together, these results demonstrate that Sensorium‐Viz effectively leverages synthetic responses to adapt learned representations, supporting cross‐mouse adaptation while reducing the reliance on extensive new recordings from each subject.

### 2.5 Individual Neurons Actively Responding to Image Properties Significantly Contribute to the Reconstructions

To better understand how neuronal activity patterns contribute to decoding, we analyzed neural signals at both the single‐neuron and population levels, examining their relationships with image properties and reconstruction metrics. Image properties were quantified using three low‐level features: spatial information (SI), which is an indicator of edge magnitudes (Yu and Winkler 2013), mean pixel intensity (brightness), and pixel intensity standard deviation (contrast). These analyses aimed to determine whether the model's reliance on neuronal activity reflects known response patterns in V1.

**FIGURE 4:**
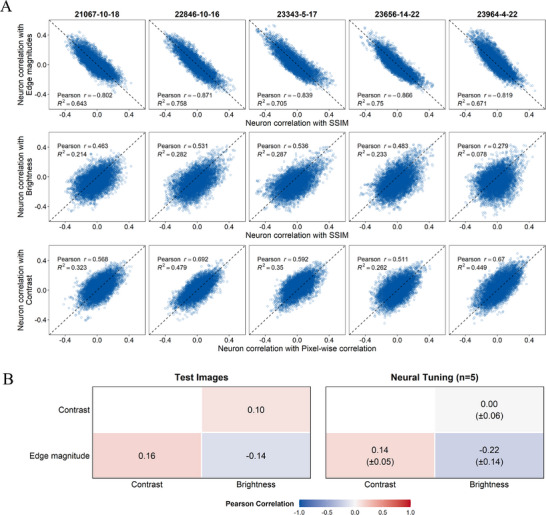
Feature‐selective neurons contribute to reconstruction quality independent of global image statistics. (A) Scatter plots correlating individual neurons' tuning strengths to image properties (*y*‐axis: Edge magnitude, Brightness, Contrast) with their contributions to reconstruction quality (*x*‐axis: SSIM or Pixel‐wise correlation). Columns represent individual mice. The Pearson r and R^2^ values indicate that individual neurons tuned to specific features are strong predictors of reconstruction quality. (B) Control analysis ruling out statistical artifacts. Left: Correlations among physical properties of the test images. Right: Aggregated cross‐neuron correlations of tuning profiles across five mice (Mean ± S.D.). The distinct structural mismatch between the image statistics (left) and the neural tuning profiles (right) confirms that the decoder relies on genuine functional heterogeneity rather than exploiting inherent visual redundancies.

At the single‐neuron level, we correlated each neuron's response with reconstruction quality metrics (SSIM and pixel‐wise correlation) across test images. Specifically, we examined whether neurons tuned to low‐level image features contributed disproportionately to decoding performance (Figure S14). Neurons exhibiting strong positive correlations with brightness and contrast also tended to show substantial positive correlations with SSIM and pixel‐wise correlation metrics (Figure 4A). Conversely, neurons sensitive to high spatial information (edge magnitude) displayed negative correlations with SSIM, consistent with a trade‐off between high‐frequency detail and structural similarity.

To verify that the strong contributions observed in Figure 4A reflected genuine biological tuning rather than statistical artifacts (e.g., collinearity between image features), we performed a control analysis examining the internal correlation structures of image properties and neural tuning profiles (i.e., whether individual neurons tuned to one feature also tend to be tuned to another; see Methods, Figure 4B). We found that physical image properties were only weakly correlated (e.g., Pearson's *r* ≈ 0.1 between contrast and brightness), and cross‐neuron tuning profiles for these features also exhibited moderate correlations. This distinct mismatch between the strong functional correlations in Figure 4A and the weak structural correlations in Figure 4B confirms that the decoder's reliance on specific neurons is not a byproduct of statistical redundancies. Instead, it indicates that the V1 network actively transforms visual information, segregating it into distinct feature‐selective channels (intrinsic functional heterogeneity) that are successfully leveraged by the decoder.

At the population level, the relationships between overall neural activity described by mean and median response amplitude, standard deviation, and skewness, and reconstruction quality were more variable and less consistent than those observed at the individual level. For instance, higher mean or median responses correlated positively with better pixel‐wise reconstruction metrics in mouse 21067, which, however, was not observed in the other mice (Figure S15). The profiles describing the distribution (skewness, mean‐median ratio, etc.) were other factors related to reconstructions. However, they also displayed heterogeneous associations with reconstruction metrics across individuals (Figure S15). These observations further emphasized that visual stimulus information may be primarily represented by distinct subsets of feature‐selective neurons rather than by global population activity patterns in V1.

## 3 Discussion

In this study, we introduced Sensorium‐Viz, a novel neuron signal decoding framework designed to reconstruct visual inputs from the primary visual cortex of mice, specifically leveraging calcium imaging data. Our approach addresses the unique challenges of decoding responses at single‐neuron levels to grayscale visual stimuli, which differ substantially from human fMRI data in previous neural decoding studies.

We first demonstrated the viability of Sensorium‐Viz by evaluating it across eight quantitative metrics. We benchmarked our approach against MinD‐Vis, which, despite being developed for human fMRI, serves as the most viable generative baseline given the lack of publicly available implementations for high‐resolution calcium imaging decoders. Notably, our method outperforms a direct application of MinD‐Vis. This is evidenced by higher scores in pixel‐wise Pearson's correlation, SSIM, and other metrics, as well as improved visual fidelity in reconstructions. This performance gap likely stems from two key mechanistic distinctions between the datasets. First, unlike the coarse, dense voxel structure of human fMRI, mouse calcium imaging is characterized by sparse, irregular single‐neuron sampling and high trial‐to‐trial variability. There are the features that MinD‐Vis's voxel‐optimized embedder struggles to capture. Sensorium‐Viz addresses this by employing an inverse distance weighting (IDW) embedder to map irregular neuron coordinates into a structured grid, thereby preserving V1 retinotopic organization. Second, to mitigate the domain mismatch between standard RGB‐trained diffusion models and the grayscale stimuli used in mouse experiments, Sensorium‐Viz incorporates a synthetic‐response augmentation strategy that compensates for limited biological data. Together, these adaptations highlight that superior performance relies on tailoring the architecture to the specific statistical irregularities and data constraints of the experimental modality.

To place these results in context, we compared our reconstruction performance with previously reported benchmarks which are in different modality or without publicly available codes and data. In human fMRI studies, pairwise similarity based on correlation was reported as 78.1%, while SSIM values reached 62.9% and 65.3% [8, 23]. By contrast, Sensorium‐Viz achieved pairwise similarity scores of 91.9% in correlation and 73.1% in SSIM. Regarding mouse calcium imaging, previous studies like Yoshida and Ohki (2020) demonstrated decoding feasibility but were limited to low‐resolution 32 × 32 outputs with median correlations of 0.43–0.45. Our method achieves a comparable median correlation of 0.46 while scaling to 256 × 256 images [15]. Notably, a concurrent study using the same Sensorium dataset, MEIcoder, reported a marginally higher pixel‐wise correlation (0.486) but a significantly lower SSIM (0.331) compared to our 0.424 [27]. This divergence highlights a fundamental trade‐off: while regression‐based methods like MEIcoder optimize pixel alignment often at the cost of blurriness, Sensorium‐Viz leverages diffusion to prioritize structural coherence, resulting in superior perceptual fidelity and sharper visual details.

Next, we explored the model's ability to generalize across different mice using synthetic data. By fine‐tuning with only synthetic responses, Sensorium‐Viz enabled a base model trained on one “source” mouse to reconstruct stimuli for a different “target” mouse. Compared to Yoshida's work [15], which reported a median correlation of 0.33 in cross‐plane experiments (i.e., different scanning areas in different mice), our method achieved a median correlation of 0.40. This number indicates that incorporating synthetic responses can support more efficient cross‐subject adaptation, potentially reducing the need for extensive new data collection.

One current limitation of this method, however, is that generating synthetic responses for a new mouse still requires an encoding model trained with some real data from that subject. This step is necessary to account for differences in the number of recorded neurons and scanned cortical regions across individuals [26]. From our scaling experiments with synthetic data, we estimate that training a decoder model fully on real data would require approximately 20 000–40 000 image–neuron signal pairs to achieve stable reconstruction performance (Figure 2). In contrast, training an encoder model to generate synthetic responses can be accomplished with 5000 or fewer real images. Although we have not yet systematically determined the minimal dataset size required, our findings suggest that synthetic data can substantially reduce—though not entirely eliminate—the need for additional recordings when adapting reconstruction models to new subjects.

To demonstrate the utility of Sensorium‐Viz for vision‐decoding studies, we examined the relationships among reconstruction quality, image properties, and neural signals at both individual and population levels. We found that individual neurons tuned to specific low‐level features, particularly brightness and contrast, were crucial predictors of reconstruction quality. In contrast, population‐level activity profiles were less predictive and highly variable across mice, suggesting that V1 encodes visual information through distinct subsets of feature‐selective neurons rather than global population patterns. Furthermore, by comparing the correlation structure of these neural tuning profiles against the statistical correlations of the images themselves, we observed a distinct mismatch. This structural mismatch confirms that the strong predictive power of these individual neurons is not a byproduct of trivial image statistics (e.g., collinear features making brighter images inherently easier to reconstruct). Instead, it indicates that V1 actively transforms visual inputs, segregating them into functionally distinct channels. Our decoder successfully leverages this intrinsic functional heterogeneity rather than exploiting statistical or population redundancies—aligning with previous findings that only a specific fraction of V1 neurons substantially drives visual representation [15, 19, 26]. While mapping the exact causal contributions of these distinct neuronal groups to the generative decoding process remains an exciting avenue for future work, our findings suggest that selectively embedding only highly predictive neurons could significantly enhance the efficiency of diffusion‐based decoders.

Another area for future improvement is reconstructing visual inputs from single‐trial or individual‐repeat data. While averaging responses across repetitions effectively mitigates the high noise inherent to calcium imaging, we acknowledge that this reliance limits the framework's practical applicability for real‐time or single‐shot decoding tasks. Our results highlight a distinct performance gap between these modalities. The absence of trial averaging leads to a marked decrease in reconstruction metrics (Figure 2C). Qualitatively, the reconstructions from single‐trial data struggle to consistently resolve the correct object features across different repeats, unlike the robust outputs derived from averaged signals (Figure S1C). Similar to our approach, the previous method that achieved high decoding accuracy also relied on the availability of repeated trials [28]. Efficiently reconstructing unseen stimuli that are shown only once therefore remains a challenge due to the noisy and variable nature of calcium signals, requiring further exploration into advanced denoising techniques or single‐shot training strategies.

In conclusion, our findings demonstrate that Sensorium‐Viz is a powerful and flexible framework for decoding visual information from mouse cortical signals. By integrating a transformer‐based diffusion model, grid‐based embedding of neuronal activity, and synthetic data augmentation, we have advanced the field's capacity to reconstruct the visual stimuli from a non‐primate visual system. This framework presents a novel in silico approach to understanding the relationship between neuronal signals in the primary visual cortex and the external visual environment.

## 4 Methods

### 4.1 Collect Image and Neuronal Response Datasets

Datasets were retrieved from the public repository: https://gin.g‐node.org/cajal/Sensorium2022. The images and neuronal response data from five mice (21067, 22846, 23343, 23656, 23964) were used to develop and evaluate the image reconstruction model. The image data consisted of grayscale images from ImageNet with a shape of 144 × 256 (144 pixels in height and 256 pixels in width). Each image would be presented to the mice for 0.5 s, followed by a black screen period between 0.3 and 0.5 s. Neuronal response data were collected from the two‐photon calcium images responding to these stimuli during the presentation periods. The signal for each neuron to each image was recorded in the accumulated relative fluorescence changes, represented by a single value. The anatomical coordinates of the neurons were also provided, representing the neuron positions relative to the pial surface. An initial partition of the train, validation, and test data was included for each mouse. The test data consisted of 100 images repeated 10 times, which were randomly mixed with the other images during the neuronal signal collection experiments.

The train/test splits are directly from the Sensorium 2022 Challenge datasets. Standard k‐fold cross‐validation was not applied due to the specific structure of the dataset, where only the designated test set contained sufficient stimulus repetitions (10 repeats) to perform the signal averaging necessary for reliable evaluation of calcium responses. Constructing comparable validation folds from the training data (which consists primarily of single‐trial) was therefore not feasible.

### 4.2 Collect Images and Generate Synthetic Neuronal Responses

Images for generating the synthetic responses were collected from the training part of the COCO 2014 Dataset (https://www.kaggle.com/datasets/jeffaudi/coco‐2014‐dataset‐for‐yolov3). The 82 784 raw images were converted to grayscale and resized to a shape of 144 × 256 to fit the input requirements of our neuronal response prediction models. We also calculated the mean and standard deviation values from the pixels of all processed images. To generate the synthetic responses, we use the winning solution that we developed for the 2022 Sensorium Challenge, available at https://github.com/GuanLab/Sensorium2022_Challenge. A set of inputs included an image, the image after centering, a bounding box of the object in the image, and the neuron positions of the mouse. This pipeline would generate 10 response predictions for each image of each mouse. The final synthetic response was obtained by averaging the 10 predicted responses, forming an ensemble output.

### 4.3 Process the Input Data for Model Training and Inference

The input data for training consisted of neuron positions and responses, along with the corresponding visual stimuli. The 1‐channel grayscale images were first converted to 3‐channel without altering colors. Each image was then resized by upscaling both sides by a factor of 256/144, followed by a center crop to produce 256 × 256 inputs. All channels were normalized with a mean and standard deviation of 0.5. No image augmentation techniques were applied, as neuron responses are sensitive to the content of the stimuli.

Neuron responses from real experiments were normalized using the same processes as in our previous work and the SENSORIUM challenges [19, 26]. We first computed the standard deviation of individual neuron responses across the entire training set, resulting in a standard deviation vector with the same length as the responses. We then element‐wise divided the raw responses by the standard deviations. Synthetic responses required no further processing, as they were generated by the model specifically designed to produce normalized responses.

Using functions from the PyInterp library, the normalized responses were further embedded into 32 × 32 grids, guided by neuron positions normalized to the range (−1, 1). Grid values were determined through the Inverse Distance Weighting (IDW) algorithm, considering 10 nearest neighbors. The resulting output matrices consisted of three channels: one for the interpolated response values, and two for the XY grid coordinates, which were evenly spaced between −1 and 1. The operations followed the PyInterp tutorials (https://cnes.github.io/pangeo‐pyinterp/auto_examples/ex_unstructured.html).

### 4.4 Model Architecture

Our model shares a similar structure to a latent diffusion model, consisting of the following components: (1) an AutoEncoder that encodes the input images to lower‐dimensional latent space and decodes the reconstructed latent representations to final output images; (2) a denoising diffusion probabilistic model (DDPM) that iteratively diffuses and denoises input images according to the guidance from the embedded responses; and (3) an encoder that encodes the information from responses to denoise the images.

#### 4.4.1 The AutoEncoder

The image data is encoded and decoded using a variational autoencoder (VAE) model with a Kullback–Leibler divergence (KL) loss, which is implemented by the AutoencoderKL module in the diffusers library. The module was loaded with the pre‐trained weights of “stability/sd‐vae‐ft‐ema” (https://huggingface.co/stabilityai/sd‐vae‐ft‐ema), which were frozen during training.

#### 4.4.2 The Response Projector Network

The architecture of this network is inspired by the input and first block layers of Attention‐Unet in the latent diffusion denoising module (Figure 1C; Figure S2A).

The network takes in the 3‐channel spatially embedded responses (shape: (3, 32, 32)), which are first encoded by a convolution layer, producing a feature map with 32 feature channels (shape: (32, 32, 32)). The resulting maps were then encoded into 64 channels through a sequential operation consisting of a group normalization with 8 groups, a SiLU nonlinearity, and a convolution layer with 64 filters. In parallel, another 64‐filter convolution layer encoded the 32‐channel input, and its output was added as a residual connection to the main branch (shape: (64, 32, 32)).

Next, a multi‐head self‐attention layer was used to encode long‐range dependencies across the spatial grid. The 64‐channel inputs were flattened (shape: (64, 32 × 32)), group‐normalized (8 groups), and passed through an attention layer with 16 heads. The outputs were then reshaped back to the original heights and widths (shape: (64, 32, 32)) and added back to the original 64‐channel inputs, forming another residual block.

Finally, the extracted feature maps were group‐normalized (8 groups), SiLU‐activated, and processed by a pointwise convolution to integrate the channel information in each grid (shape: (1, 32, 32)). The processed maps were then flattened into vectors and passed through a linear layer, producing conditioning vectors with a length of 1152 that represented the entire neuronal response pattern. The dimensionality of 1152 matches the predefined hidden size of the DiT‐XL/2 model.

#### 4.4.3 The Denoising Diffusion Probabilistic Model (DDPM)

Briefly, a DDPM consists of two steps: a forward diffusion process and a learnable denoising process. During the forward diffusion process, small Gaussian noise is gradually added to the real data over *T* timesteps. Mathematically, given a real data point *x*_0_, and with the reparameterization trick, the noisy data *x_t_* at the timestep *t* can be expressed as:

(1) $$\;x_{t}=\sqrt{{\bar{\alpha }}_{t}}\;x_{0}+\sqrt{1-{\bar{\alpha }}_{t}}\varepsilon_{t},$$

where ${\bar{\alpha }}_{t}=\prod_{s=1}^{t}\;\alpha_{s}$, and $\varepsilon_{t}∼N\left(0,I\right)$

In implementation, α_*s*_ is calculated from 1‐β_*s*_, where β_*s*_ is retrieved from a linear space with 1000 data points from 0.0001 to 0.02 (a linear scheduler with 1000 diffusion timesteps). As *T*  →  ∞, *x_T_* becomes equivalent to a point sampled from an isotropic Gaussian distribution.

During the reverse denoising process, a denoising function is trained to learn a parameterized distribution that approximates the reverse of the forward process, enabling the recovery of clean samples from noisy inputs. Given *x_t_*, this process estimates the conditional distribution of *x*_*t*‐1_ at the previous timestep:

(2) $$p_{\theta }\left(x_{t-1}|x_{t}\right)=N\left(x_{t-1};\mu_{\theta }\left(x_{t},t\right),\Sigma_{\theta }\left(x_{t},t\right)\right)$$

where μ_θ_ and Σ_θ_ are statistics that can be learned by a neural network, which, in our method, is the diffusion transformer (DiT). In practice, one way to estimate *p*_θ_ in DDPM is training a neural network ε_θ_(*x_t_*,*t*) to approximate the real noise ε_*t*_ added at the timestep *t* instead of μ_θ_. Once *p*_θ_ trained, new images can be sampled by initializing $x_{tmax}∼N(0,\;I)$ and repeatedly sampling $x_{t-1}∼p_{\theta }(x_{t-1}|x_{t})$.

#### 4.4.4 The Diffusion Transformer (DiT)

DiT shares a similar network structure as the ViT, including a patch embedder (patch size = 2) and a sin‐cos position embedder to patchify the inputs—the latent images encoded by the autoencoder—into image tokens. These tokens are then processed by 28 DiT blocks, which incorporate conditioning information from the neuron responses and timesteps. A final layer decodes the tokens and unpatchifies them to produce two outputs: a noise prediction and a diagonal covariance prediction. Both outputs have the same shape as the input.

Within the DiT blocks, the timestep is encoded by a sinusoidal timestep embedder and added to the encoded neuron response information, forming the conditions that guide the current denoising step. Conditions were incorporated into the blocks through an adaptive layer norm (adaLN)‐Zero module. Inside this module, a linear encoder maps the conditions to six tensors: (α_1_,β_1_,γ_1_,α_2_,β_2_,γ_2_). Each has the same shape as the input conditions. The six tensors are applied to a transformer block through scale and shift operations. Mathematically, given an input *x*, a multi‐head self‐attention layer *MSA*, and a point‐wise feed‐forward layer *MLP*, the operation in one block can be written as the following, ignoring the normalization steps:

(3) $$x_{\mathrm{msa}}=x+\alpha_{1}\mathrm{MSA}\left(x\cdot \left(1+\gamma_{1}\right)+\beta_{1}\right)$$

(4) $$x_{\mathrm{out}}=x_{\mathrm{msa}}+\alpha_{2}\mathrm{MLP}\left(x_{\mathrm{msa}}\cdot \left(1+\gamma_{2}\right)+\beta_{2}\right)$$

All the multiplications in these formulas are element‐wise. A similar conditioning step was also applied in the final layer, where γ and β were generated to scale and shift the inputs.

Where γ and β were generated to scale and shift the inputs.

### 4.5 The Training Losses

The model is trained to optimize both the predicted noises and the learned reverse process covariance. The loss between the real Gaussian noise and the prediction at the timestep *t* is defined by a mean squared error (MSE):

(5) $$L_{\mathrm{simple}}={\left\|\varepsilon_{\theta }\left(x_{t},t\right)-\varepsilon_{t}\right\|}_{2}^{2}$$

For the covariance, the model minimizes the following variational lower bound loss given by the KL‐divergence *D_KL_* between the true posterior distribution defined by the forward process *q* and the learned reverse process *p*_θ_ at *t* ≥ 1:

(6) $$L_{\mathrm{vlb}}=D_{\mathrm{KL}}\left(q\left(x_{t-1}|x_{t},x_{0}\right)\;∥\;p_{\theta }\left(x_{t-1}|x_{t}\right)\right)$$

Since both *q* and *p*_θ_ are Gaussian, the divergence can be calculated from the mean and variance of these two distributions (μ(*t*), Σ(*t*), μ_θ_(*t*), Σ_θ_(*t*)). Using the α,  β defined in the previous DDPM section (Equation 1), we can derive the posterior mean μ and log variance Σ by:

(7) $$\mu \left(t\right)=\frac{\beta_{t}\sqrt{1-{\bar{\alpha }}_{t-1}}}{1-{\bar{\alpha }}_{t}}\;x_{0}+\frac{\left(1-{\bar{\alpha }}_{t-1}\right)\sqrt{\alpha_{t}}}{1-{\bar{\alpha }}_{t}}x_{t}$$

(8) $$\Sigma \left(t\right)=\log\left(\frac{\beta_{t}\left(1-{\bar{\alpha }}_{t-1}\right)}{1-{\bar{\alpha }}_{t}}\right)$$

The mean μ_θ_(*t*) of *p*_θ_ can be calculated from the learned noise ε_θ_(*t*):

(9) $${\hat{x}}_{0}=\sqrt{\frac{1}{{\bar{\alpha }}_{t}}}\;x_{t}-\sqrt{\frac{1}{{\bar{\alpha }}_{t}}-1}\;\varepsilon_{\theta }\left(t\right)$$

(10) $$\mu_{\theta }\left(t\right)=\frac{\beta_{t}\sqrt{1-{\bar{\alpha }}_{t-1}}}{1-{\bar{\alpha }}_{t}}\;{\hat{x}}_{0}+\frac{\left(1-{\bar{\alpha }}_{t-1}\right)\sqrt{\alpha_{t}}}{1-{\bar{\alpha }}_{t}}\;x_{t}$$

while the log variance Σ_θ_(*t*) is computed from the covariance output $\Sigma_{\theta }^{*}(t)$ of the final DiT layer:

(11) $$\Sigma_{\theta }\left(t\right)=\frac{\Sigma_{\theta }^{*}\left(t\right)+1}{2}\;\log\left(\beta_{t}\right)+\left(1-\frac{\Sigma_{\theta }^{*}\left(t\right)+1}{2}\right)\Sigma \left(t\right)$$

when *t*  =  0, *L_vlb_* reduces to ‐*p*(*x*_0_|*x*_1_), which corresponds to the log‐likelihood of a Gaussian distribution, which is parameterized by μ_θ_(1), and 0.5*Σ_θ_(1), discretizing to *x*_0_. The total loss used to train the model is given by *L*  = *L_simple_*  +*L_vlb_*.

### 4.6 The Classifier‐Free Guidance (CFG)

Conditional diffusion models take extra input, such as embedded text, labels, or, in our case, neuron response, as guidance for image generation. To encourage the model to better understand and rely on these conditions, previous works employ a technique known as classifier‐free guidance, in which some samples are intentionally created without including the condition during training. This method teaches the model to distinguish between conditioned and unconditioned outputs, allowing it to use the guidance more effectively when it is provided during generation. This technique is widely used and generally yields significantly better sample qualities. In the image‐class‐guided generation, a CFG sample has an additional “null” class other than the actual classes.

To utilize the CFG technique in our neuron signal‐guided reconstruction, we created “null” responses by setting the first embedded response channel (the channel for neuron responses) to 0 while keeping the position information in the other two channels. The dropout rate was 0.1, meaning that 10% of the training data in each batch were assigned “null” responses.

### 4.7 Devices and Settings for Training the Models

The models were trained on one or multiple NVIDIA L40S 48G GPUs, with a batch size of 32 per GPU and 80,000 training steps. The parallel training across multiple GPUs was handled by the “DistributedDataParallel” (DDP) module of PyTorch. Losses were optimized by the “AdamW” optimizer with a learning rate of 5e‐5, default Adam betas (0.9, 0.999), and 0 weight decay. To stabilize the training process, a gradient clipping step was introduced during weight updates, which limits the gradient norm of the parameters to a maximum of 1.0 (max_norm = 1.0).

Our preliminary experiments observed abnormal loss changes, where the losses could suddenly increase and explode to NaN, which may be due to outliers in both the real and synthetic datasets. To eliminate these samples, we trained a model with all samples from the five mice and monitored the loss changes by the exponential moving average (EMA) of the losses. Batches with suspected outliers would be saved when the loss exceeded three times the EMA or became NaN. A total of 1809 outliers (1694 synthetic and 115 real) were excluded from the training set.

### 4.8 Reconstruction With the Neuron Responses

After training a model, we generate the reconstructions from random noises in the latent space and under the guidance of the embedded neuron responses. The noises had the shape of (4, 32, 32), where “4” is the channel number and the latter two values represent the width and height. These numbers are the shapes of the auto‐encoder outputs.

We also applied the CFGs in the inference stage. The initial latent image would be repeated twice to generate reconstructions under real and null conditions, and the input conditions also consisted of real and null neuron responses, as prepared above. The output of the model (ε_θ_) which estimates the real noise added at the timestep *t* in the diffusion steps, would be derived from the unconditioned (ε_θ, *uncond*_) and conditioned (ε_θ, *cond*_) estimations with a CFG scale (4.0 in our implementation):

(12) $$\varepsilon_{\theta }=\varepsilon_{\theta ,\mathrm{uncond}}+\mathrm{CFG}\cdot \left(\varepsilon_{\theta ,\mathrm{cond}}-\varepsilon_{\theta ,\mathrm{uncond}}\right)$$

Then, with the mean and log variance we derived from Equations (10) and (11), we gradually generated the reconstruction by:

(13) $$x_{t-1}=\mu_{\theta }\left(t\right)+\exp\left(0.5\Sigma_{\theta }\left(t\right)\right)\varepsilon ,\;\varepsilon ∼N\left(0,I\right)$$

The noise term will be removed when *t*  =  0.

### 4.9 Evaluation

To ensure the robustness and generalizability of our results in the absence of cross‐validation, we adopted a multi‐level validation strategy: (1) Biological Replication: We trained and evaluated five independent models corresponding to the five distinct mice (Table 1), treating each subject as a biological replicate. (2) Inference Stability: For every test image, we generated four reconstructions using different random seeds during the reverse diffusion process. We reported the mean and standard deviation across these generations to quantify the variance inherent to the generative model.

The performance of reconstructions is evaluated using both pixel‐level and overall image content metrics, including mean squared errors (MSE), Pearson's pixel‐wise correlation, learned perceptual image patch similarity (LPIPS), and structural similarity index (SSIM). All the metrics receive a pair of images (the ground truth  *I_gt_* and the reconstruction  *I_recon_*) as inputs, where the pixel values range from 0 to 1. A matrix of Gaussian noise with the same shape as *I_recon_* is used to calculate the random baseline of each metric. The baseline values for the metrics are around 0.14 (MSE), 0 (pixel‐wise correlation), 0.846 (LPIPS), and 0.007 (SSIM), respectively. The baseline values for the pairwise similarities are all 0.5.

MSE and pixel‐wise correlation are measured pixel by pixel and implemented using their formulas and the functions from the Python NumPy package. SSIM is implemented with the “structural_similarity” function from scikit‐image [29]. The “data_range” parameter in this function is set to the difference between the maximum and minimum pixel values  *I_recon_*. LPIPS computes the similarity between the activations of images from a predefined network, which is implemented using “LearnedPerceptualImagePatchSimilarity” from TorchMetrics with a pre‐trained VGG‐16 network (net_type = “vgg”) [30]. Additionally, we employ pairwise similarity comparisons based on these metrics to assess accuracy, as used in previous research. For an inference, it will be compared with the other gold standards, and the metrics will be calculated (Figure 2B). The pairwise similarity measures how specific the inferences are reconstructed for the gold standard.

### 4.10 Image Properties

We extracted three low‐level image properties: edge magnitude, brightness, and contrast. For grayscale images, brightness was calculated as the mean pixel intensity, and contrast as the standard deviation of pixel intensities. Edge magnitude was quantified using Spatial Information (SI) (Yu and Winkler 2013).

Specifically, SI was computed from the Sobel filter responses. For each pixel, the gradient magnitude is $\sqrt{S_{h}^{2}+S_{v}^{2}}$, where *S*_h_ and *S_v_* denote responses to horizontal and vertical Sobel filters, respectively. The final SI score is the average of these magnitudes across the image.

### 4.11 Calculation of Neuronal Tuning Scores and Cross‐Neuron Correlations

To quantify the functional selectivity of individual neurons and the correlation structure of these selectivities across the network, we performed the following analyses on the test set:

#### 4.11.1 Image Feature Vectors

For each test image *k*, we computed three scalar properties: Brightness (mean pixel intensity), Contrast (standard deviation of pixel intensities), and Edge Magnitude (Spatial Information, SI). This yielded three feature vectors (e.g., *V*_*Brig*h*ness*_), each containing the property values across the 100 distinct test images.

#### 4.11.2 Single‐Neuron Tuning Scores

For each neuron *n*, we defined its “tuning score” to a specific feature as the Pearson correlation coefficient between the neuron's average response vector (*R_n_*) and the corresponding image feature vector. For example, the brightness tuning score for neuron *n* is calculated as *r*_*n*, *brig*h*t*_ =  *corr*(*R_n_*, *V*_*Brig*h*tness*_). These scores (y‐axis in Figure 4A) represent how strongly and linearly a specific neuron modulates its firing rate in response to variations in a low‐level image feature.

#### 4.11.3 Cross‐Neuron Tuning Correlations

Figure 4B, To examine the relationship between different functional selectivities across individual neurons, we constructed “tuning profile vectors” by aggregating the tuning scores of all recorded neurons (e.g., *T*_*Brig*h*tness*_ =  [*r*_1, *brig*h*t*_,  …,  *r*_*N*, *brig*h*t*_], where *N* is the number of neurons). The values reported in the neural panels of Figure 4B represent the Pearson correlation coefficients between these population tuning vectors (e.g., *corr*(*T*_*Brig*h*tness*_, *T_Contrast_*)). A negative correlation implies that neurons strongly tuned to one feature (e.g., brightness) tend to be weakly or negatively tuned to the other (e.g., contrast).

### 4.12 Application of MinD‐Vis to Mouse Neuron Responses

To compare our model with current state‐of‐the‐art techniques for reconstructing visual stimuli from brain signals, we applied the techniques used in MinD‐Vis to neuron responses [10].

MinD‐Vis is a latent diffusion model trained in two separate stages. The first stage, referred to as “sparse‐coded masked brain modeling” (SC‐MBM), involves training a masked autoencoder to reconstruct masked signals and embedding the input neuron signals in a latent space. Our input neuron responses, both real and synthetic, were ordered by their anatomical positions as provided in the raw data. The mask ratio was 0.75. To ensure non‐negative reconstructions, we wrapped the original output in an ELU activation with a +1 offset. All the other settings were kept the same as MinD‐Vis suggested. We trained for 400 epochs using the standard MSE loss between the gold standard and reconstructed responses on the training data. We then fine‐tuned the embeddings on the test responses for an additional 25 epochs.

The second stage, referred to as “double‐conditioned latent diffusion modeling” (DC‐LDM), utilizes the learned encoder from the first stage as conditioning to fine‐tune a latent diffusion model. We also followed the same settings as MinD‐Vis and trained on our datasets for 280 epochs for each mouse. We saved the model every 20 epochs. The weights that achieved the best pixel‐wise correlation were loaded for benchmarking and making inferences on each mouse.

### 4.13 Statistical Tests

All statistical *p*‐values reported in the results were calculated using the Wilcoxon signed‐rank test, implemented via the *wilcox.test* function in the R programming language.

Comparisons between Sensorium‐Viz and MinD‐Viz were conducted on $n = 20$ data points, comprising five mice (IDs: 21067, 22846, 23343, 23656, 23964) with 4 independent inference repetitions (random seeds) per mouse. P‐values were derived from one‐sided paired Wilcoxon tests to determine whether the performance of Sensorium‐Viz was significantly higher than that of MinD‐Viz.

Statistical comparisons for the cross‐mouse inference performance against MinD‐Viz were conducted between the cross‐mouse results (*n* = 24; 6 source‐target combinations × 4 repetitions) and the MinD‐Vis results for the corresponding target mice (n = 12; 3 subjects × 4 repetitions). These comparisons utilized unpaired one‐sided Wilcoxon rank‐sum tests (setting paired = FALSE) to assess significance.

## Conflicts of Interest

The authors declare no conflict of interest.

## Code Availability

The codes of this work are available at: https://github.com/GuanLab/sensorium‐viz.

## Supporting information




**Supporting File**: advs74601‐sup‐0001‐SuppMat.pdf

## Data Availability

Datasets of the mice can be retrieved from https://gin.g‐node.org/cajal/Sensorium2022. Synthetic responses for the COCO images and the model weights needed to reproduce the paper results can be obtained from Google Drive: https://drive.google.com/drive/folders/1GbJ7V2AzVezKW3U0lwhrKYaeQni2ntef.
