# Supplementary material for: Learnable Diffusion Framework for Mouse V1 Neural Decoding
Source: Adv Sci (Weinh). 2026 Mar 5;13(30):e20220. doi: 10.1002/advs.202520220 (PMC13248838; doi:10.1002/advs.202520220)

Supplementary Figure 1: Comparing the reconstruction qualities of our method with MinD-Vis. The reconstructions are from preliminary experiments that do not include synthetic samples or repetition merging. Images in (A) are from MinD-Vis with its recommendation parameter settings and training pipelines, while (B) are the results from MinD-Vis where we unfreeze the weights of the diffusion module for training. (C) contains the results of our method. The first column of all image grids is the ground truth. The remaining columns are the repeating generations starting from different random noises.

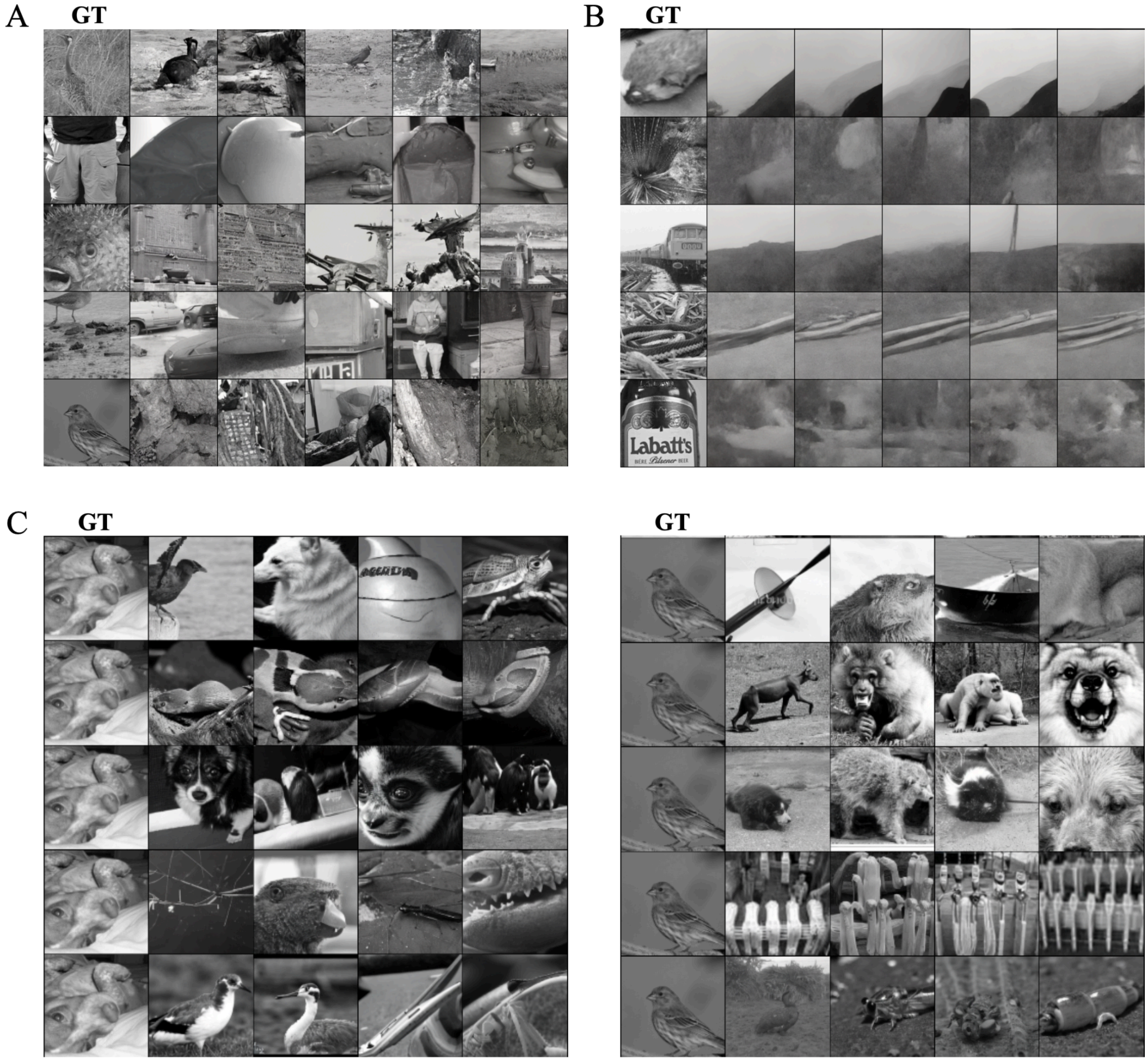

Supplementary Figure 2: The detailed structure of the response projector and DiT blocks with conditions. We use "#" to abbreviate "the number of" in this figure. (A) presents the layers with parameters for the response projector. The "c", "k", "s", and "p" in the convolution layers (Conv2d) represent the output channel number, kernel size, stride size, and padding size, respectively. The two numbers in the parentheses in the Linear layer represent the input feature number and the output feature number. (B) shows the layers of the DiT blocks, modified from the original DiT paper (Peebles and Xie 2022). The parentheses in the Linear layers indicate the number of input and output features. "Pointwise Feedforward" consists of two stacked linear layers, which first project the inputs to a four times larger hidden layer and then project them back to their original size. "Shift" and "Scale" are two special operations in the adaLN-Zero conditioning. The formulas on the right show how the input data is calculated with these operations and coefficients.

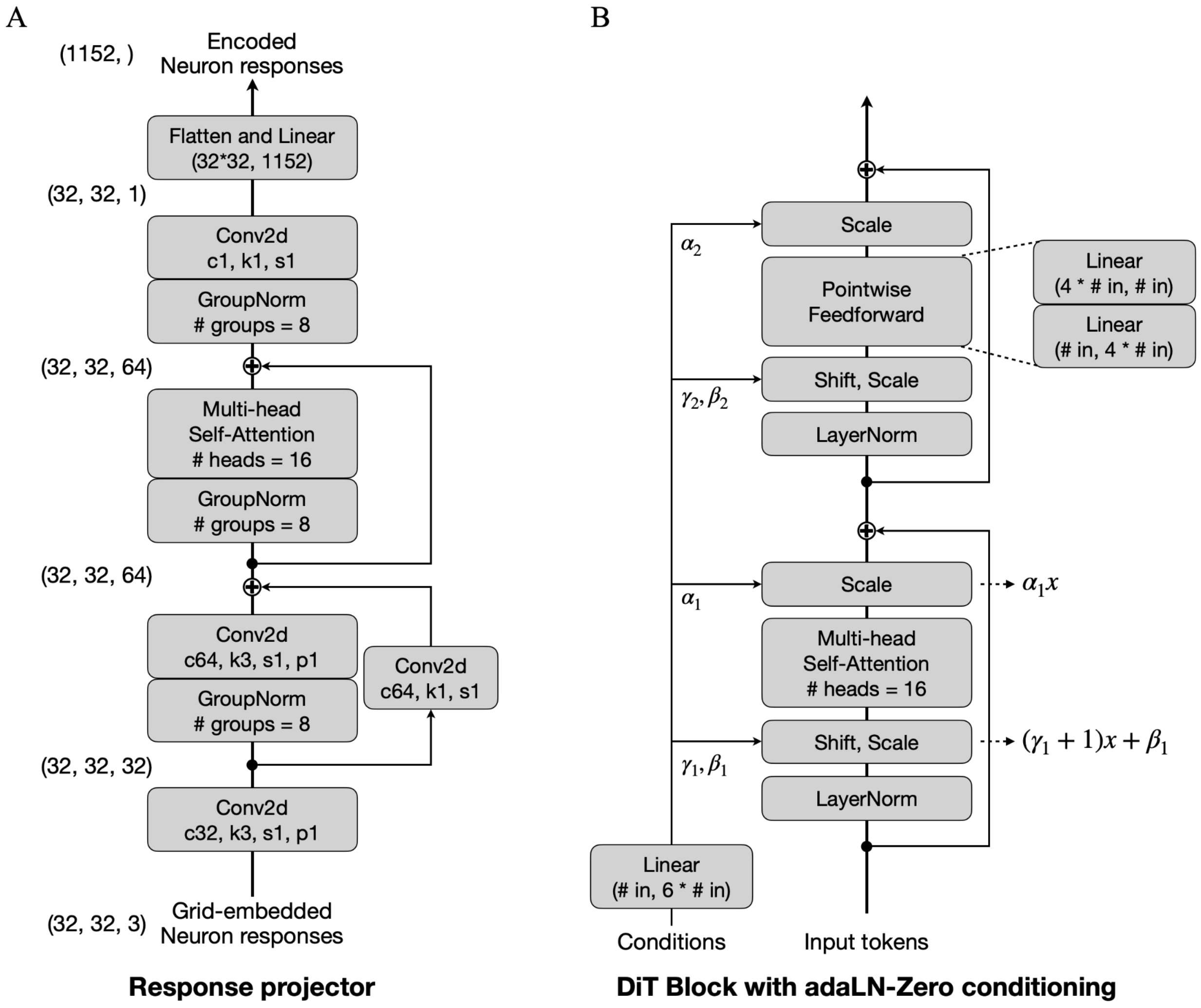

Supplementary Figure 3: Examples of the signal correlations among the repetitions comparing within and across the instances. The five panels of this figure are the results for the five mice. The upper eight images are repeated instances where we check the signal correlations. The heatmaps in the lower parts consist of two sections: the lower triangle areas are the correlations from the raw neuron responses, while the upper triangular areas are for the grid-embedded responses, separated by the white squares on the diagonals. The white boxes surround the tiles in which the repetitions are from the same instances.

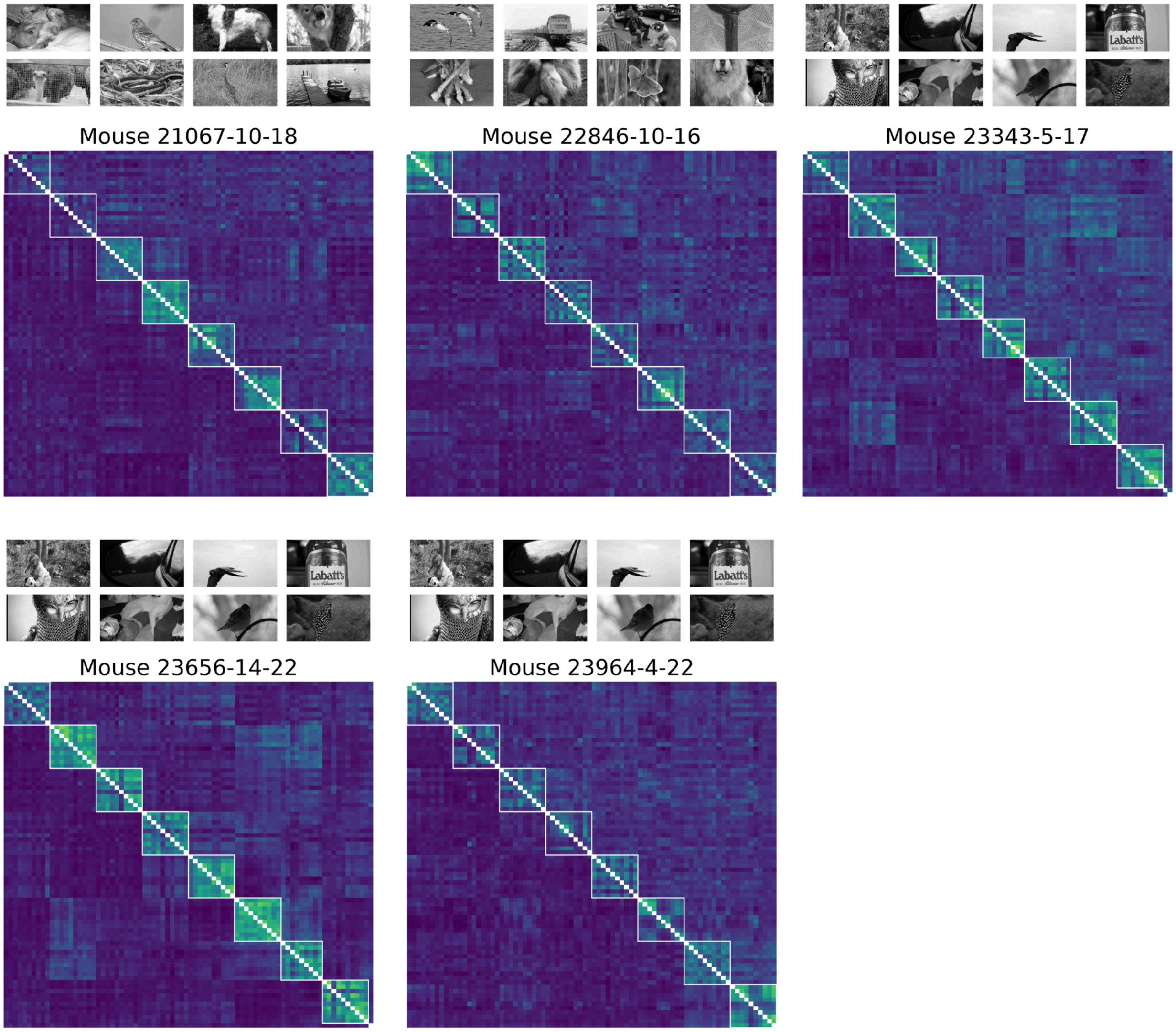

Supplementary Figure 4: Results of signal correlation under different interpolation parameters. Here, we investigate and visualize signal correlations under different grid sizes (32, 64), interpolation methods (IDW, RBF), and kernels for the RBF method (linear, Gaussian). (A) shows the average correlations comparing repeats within and across test instances, as shown in Figure 5.1E. (B) shows the heatmaps of the signal correlations for the first 10 test instances in mice 23343 and 23656. All the results are from the embedded signals. We exclude the values on the diagonals in these plots.

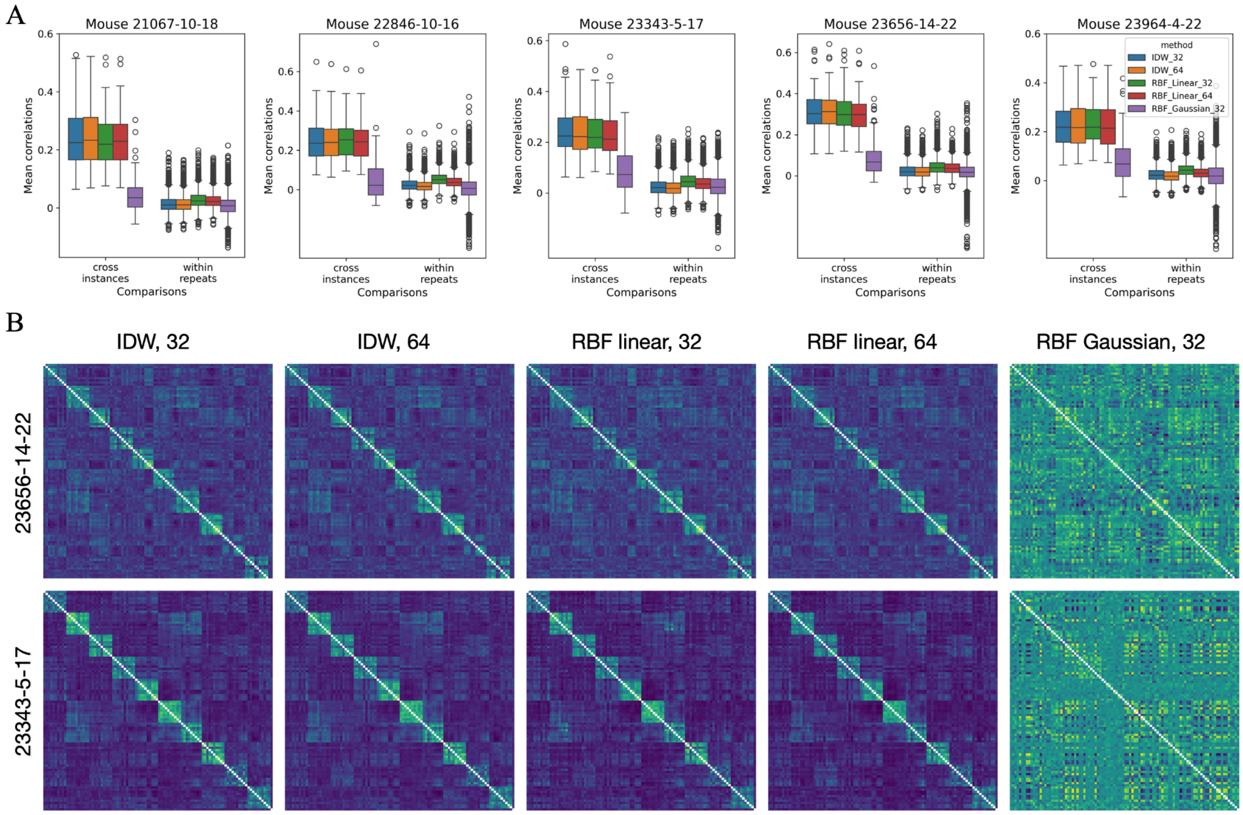

Supplementary Figure 5: Examples of MinD-Vis SC-MBM embedder. The plots are generated from the wandb logger used in the MinD-Vis codes. The x-axes are the neurons ordered by their 3D-position (X, Y, Z) values from the minimum to the maximum. The first column shows the original neuron responses. The second column is the masked responses with a mask ratio of 0.75 for encoding. The third column is the recovered response from the SC-MBM decoder. Correlations between the original and recovered responses are listed on the right.

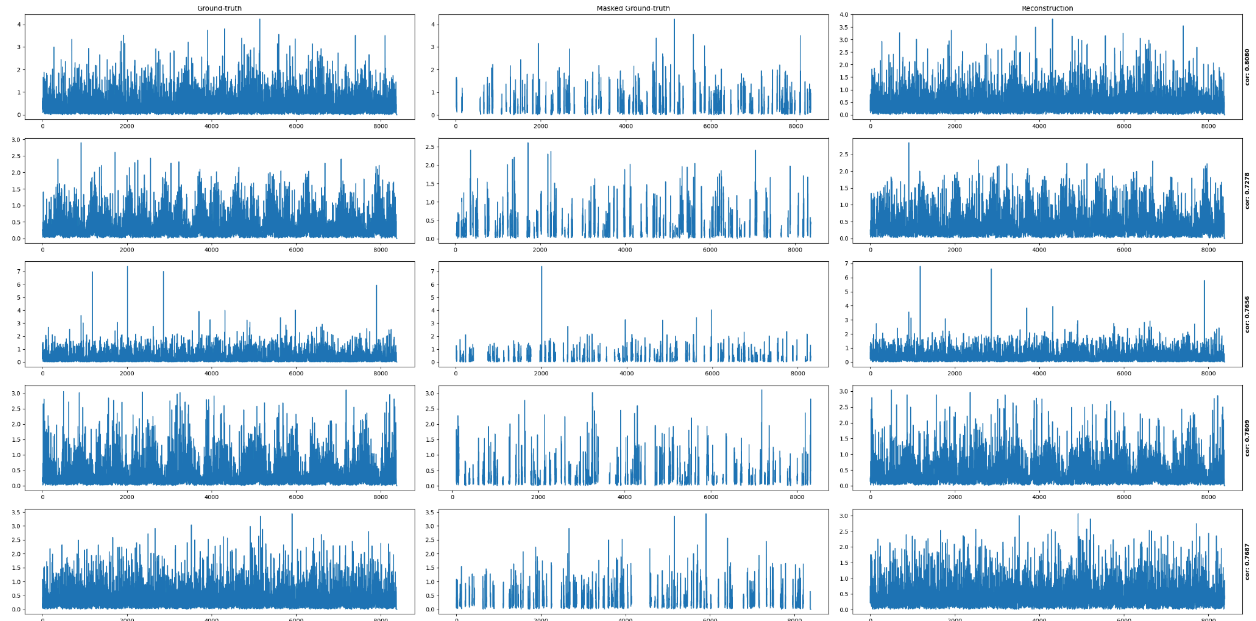

Supplementary Figure 6: The MSE and LPIPS results for comparing synthetic sample numbers. This plot corresponds to Figure 5.2C. The metric scores and their pairwise similarities from all mice are visualized under different numbers of synthetic samples.

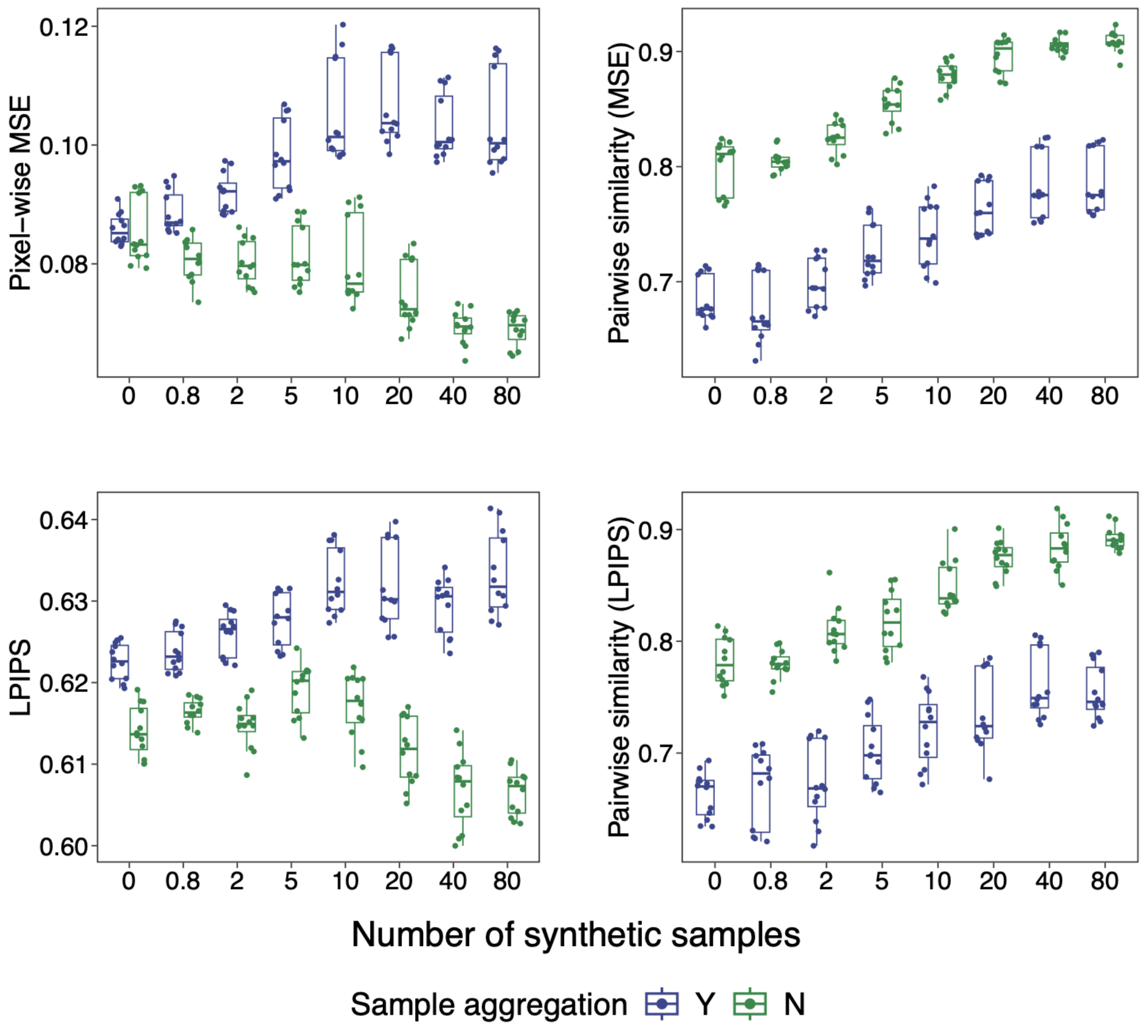

Supplementary Figure 7: The MSE and LPIPS results for comparing methods. This plot corresponds to Figure 5.3A.

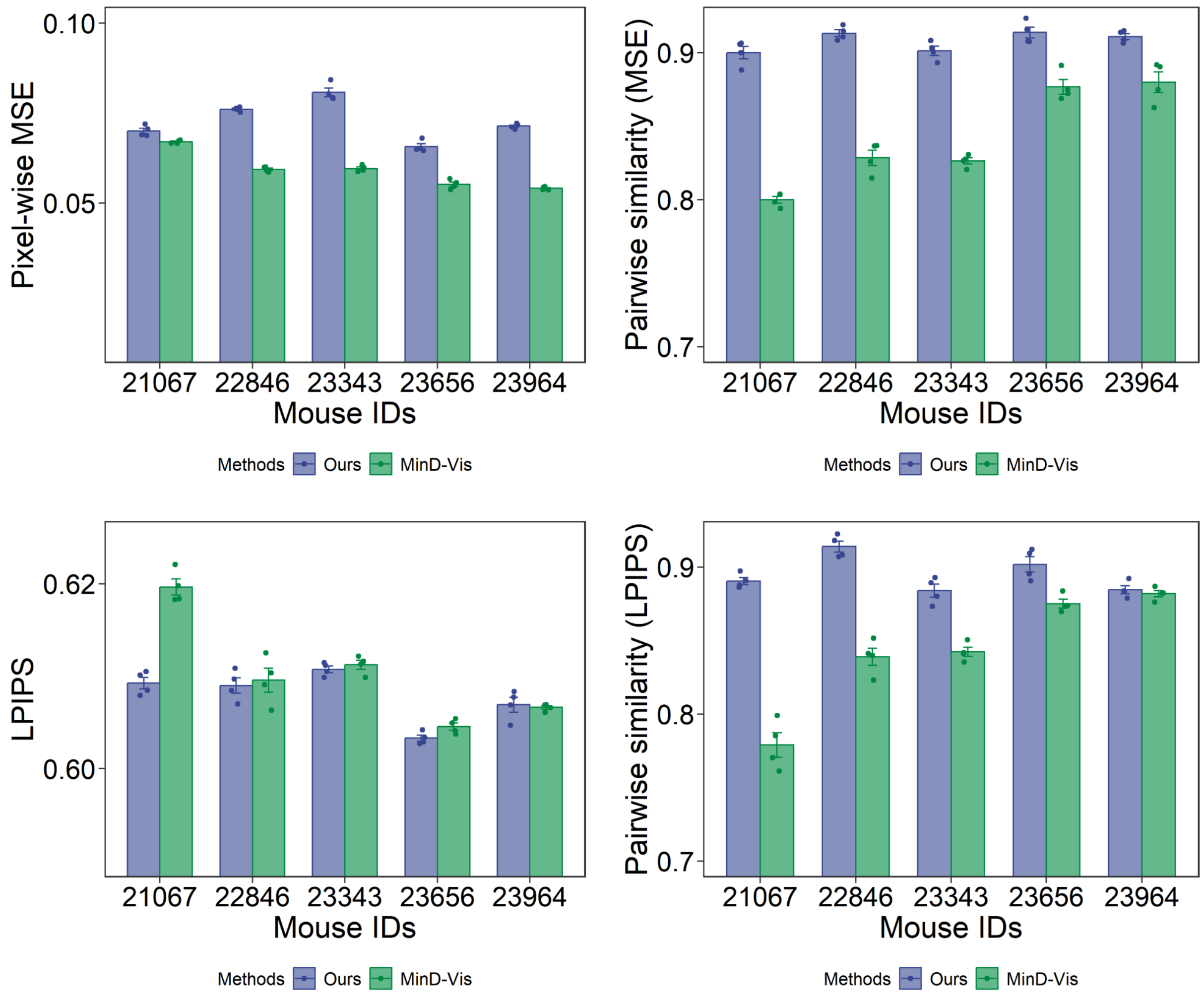

Supplementary Figure 8: The MSE and LPIPS results for cross-mice predictions. This heatmap corresponds to Figure 5.3C. The x-axis labels are the mice providing the base model, while the y-axis labels are the mice providing the synthetic data for fine-tuning.

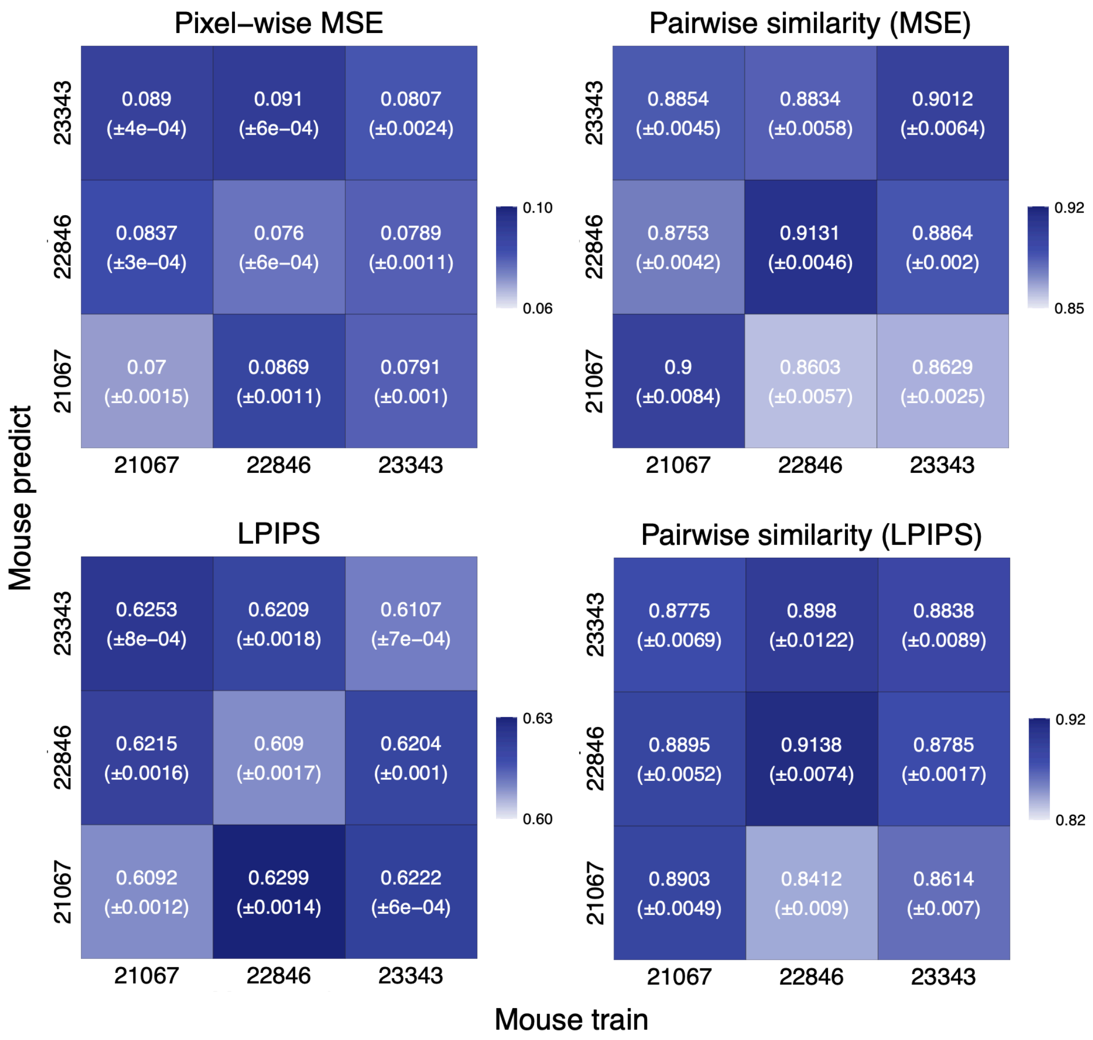

Supplementary Figure 9: The full reconstruction results of mouse 21067. The reconstructions from both methods are based on models trained with 80,000 synthetic responses and merged neuron responses. Images are sorted by LPIPS scores and compared to MinD-Vis.

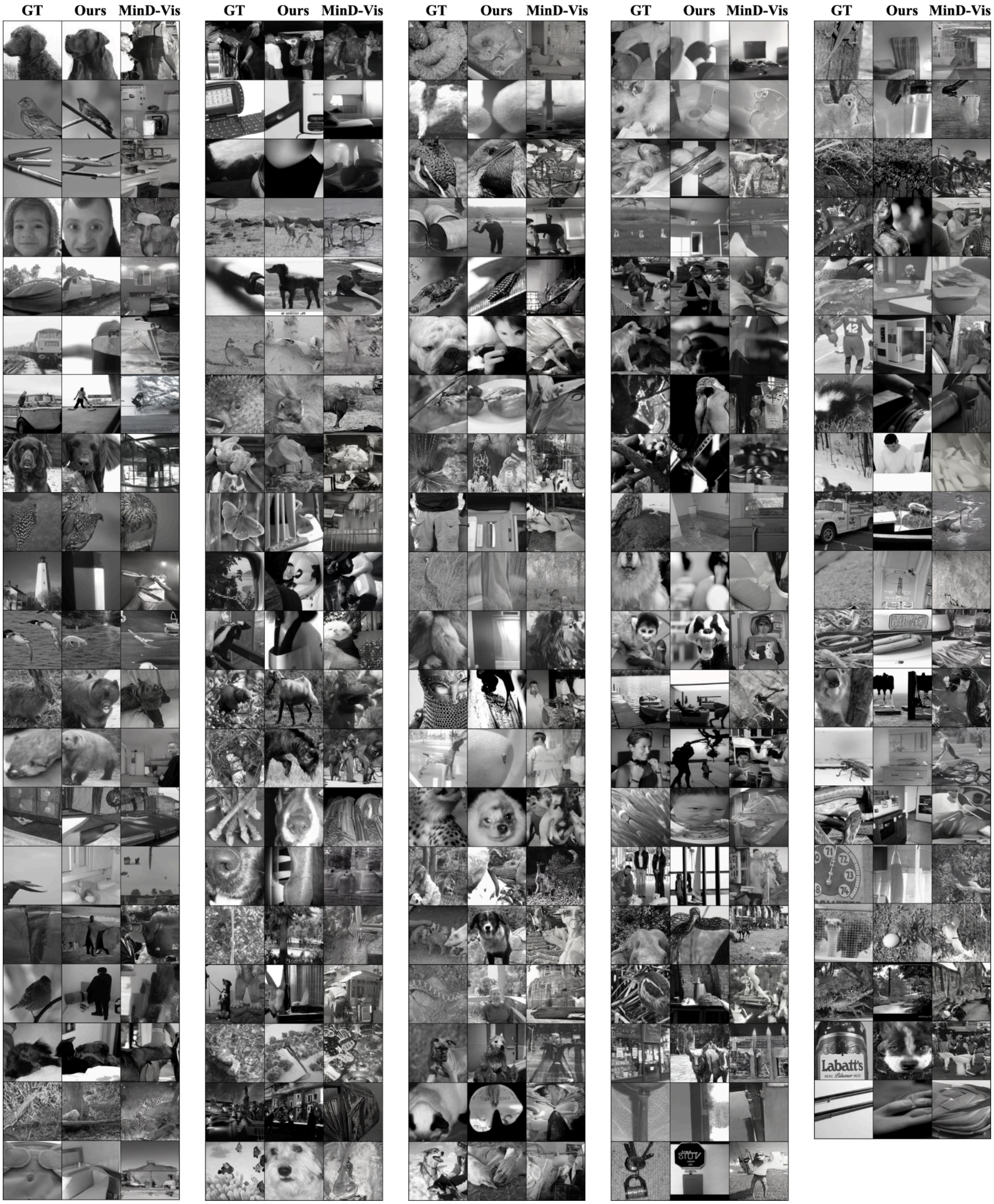

Supplementary Figure 10: The full reconstruction results of mouse 22846. Images are sorted by LPIPS scores and compared to MinD-Vis.

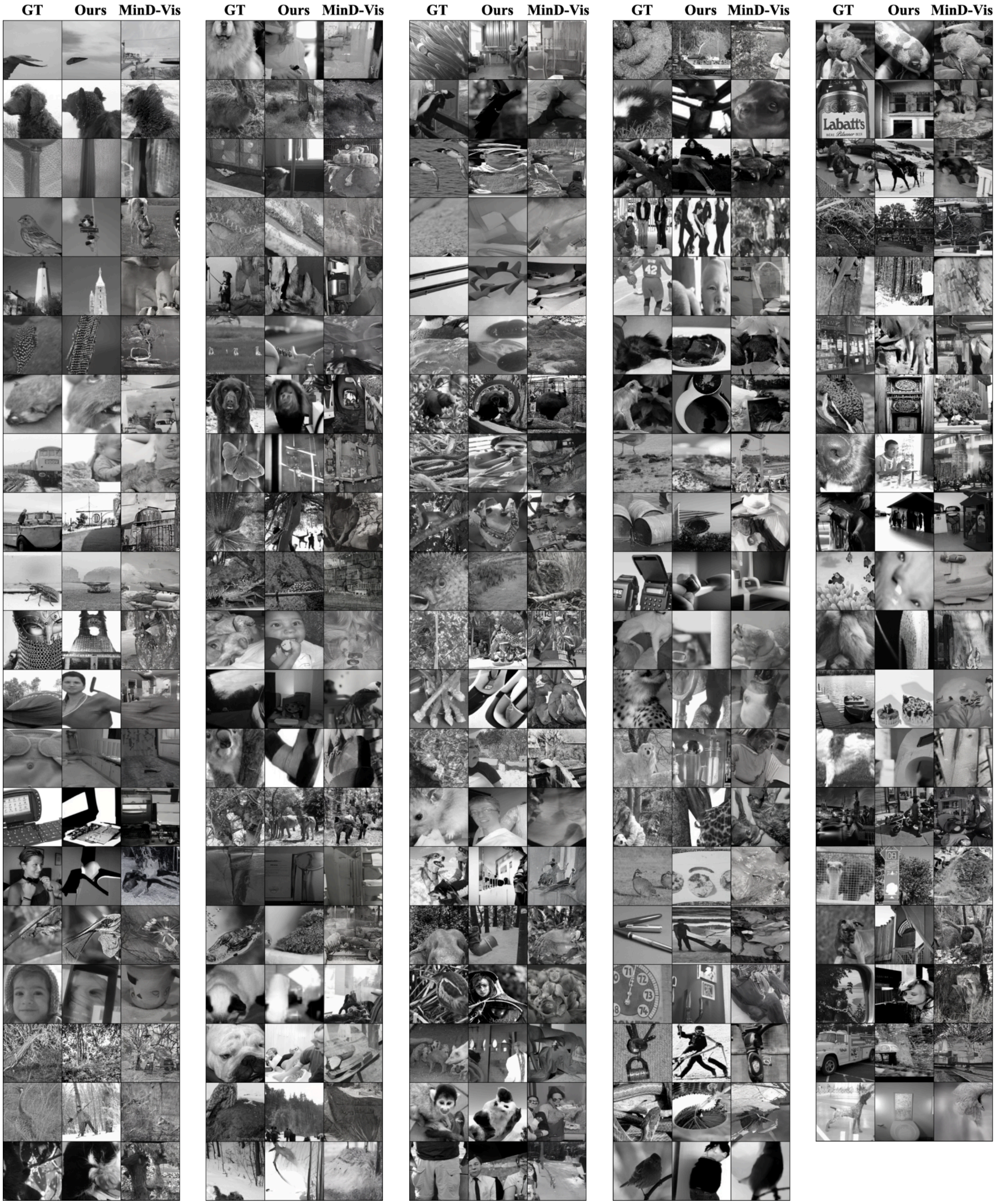

Supplementary Figure 11: The full reconstruction results of mouse 23656. Images are sorted by LPIPS scores and compared to MinD-Vis.

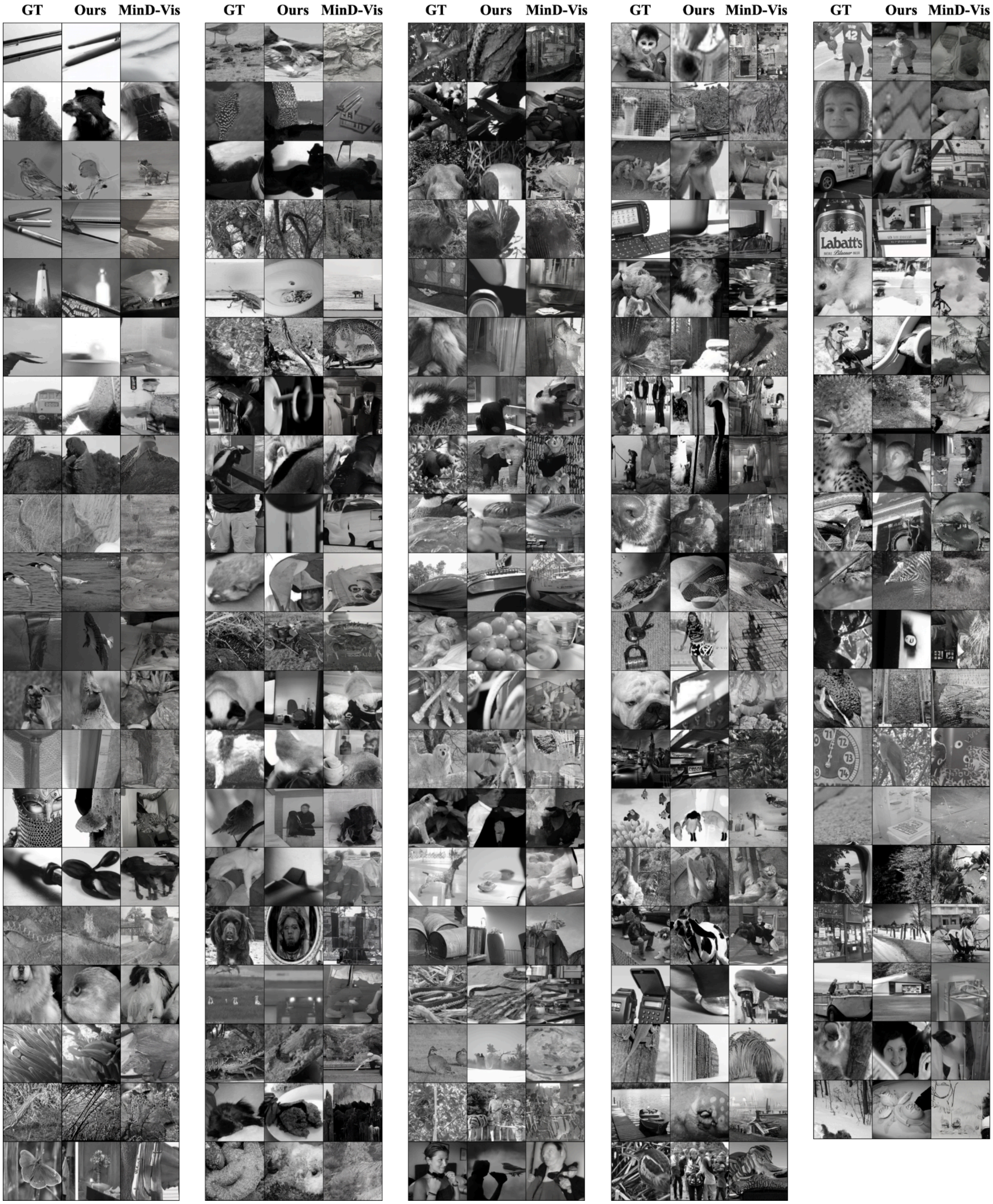

Supplementary Figure 12: The full reconstruction results of mouse 23343. Images are sorted by LPIPS scores.

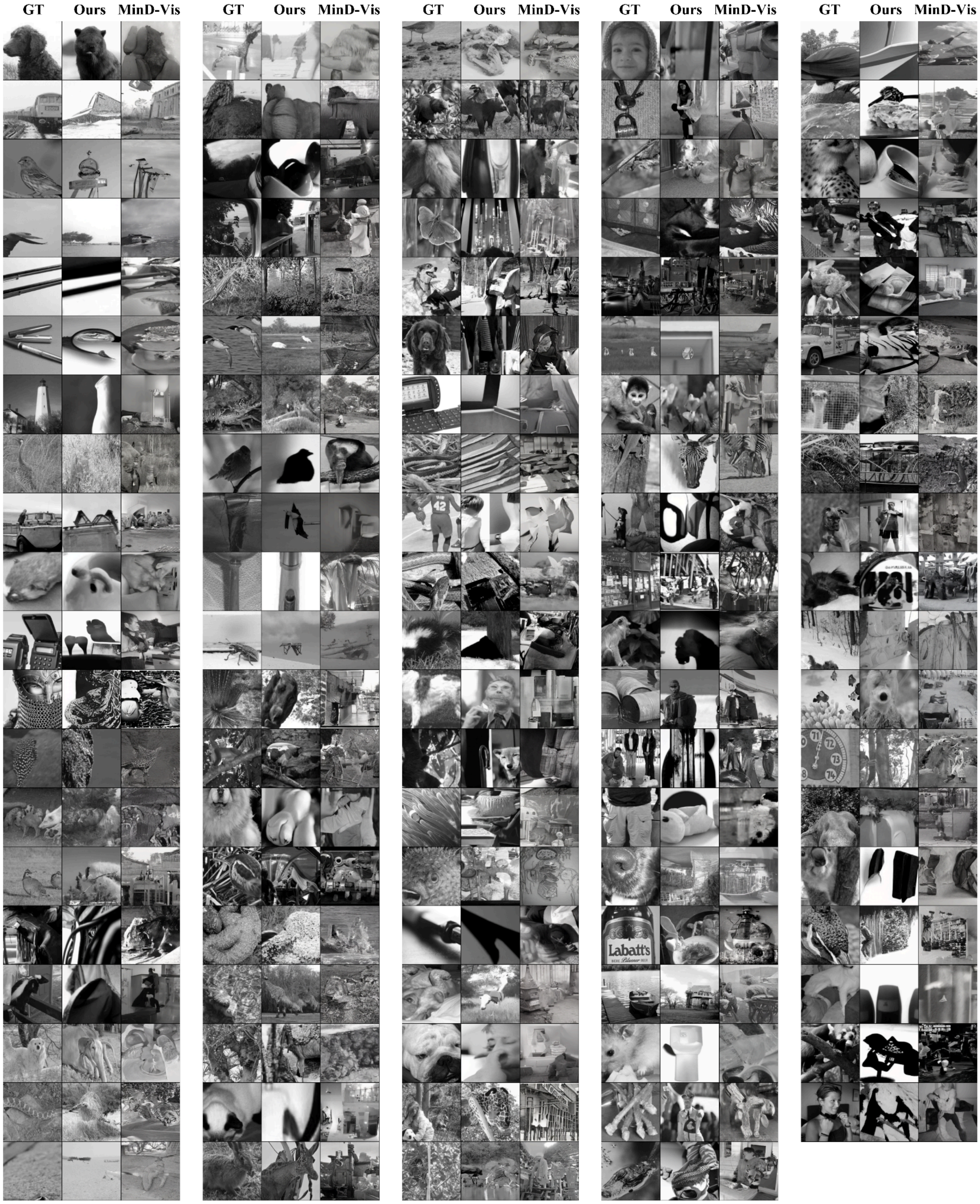

Supplementary Figure 13: The full reconstruction results of mouse 23964. Images are sorted by LPIPS scores.

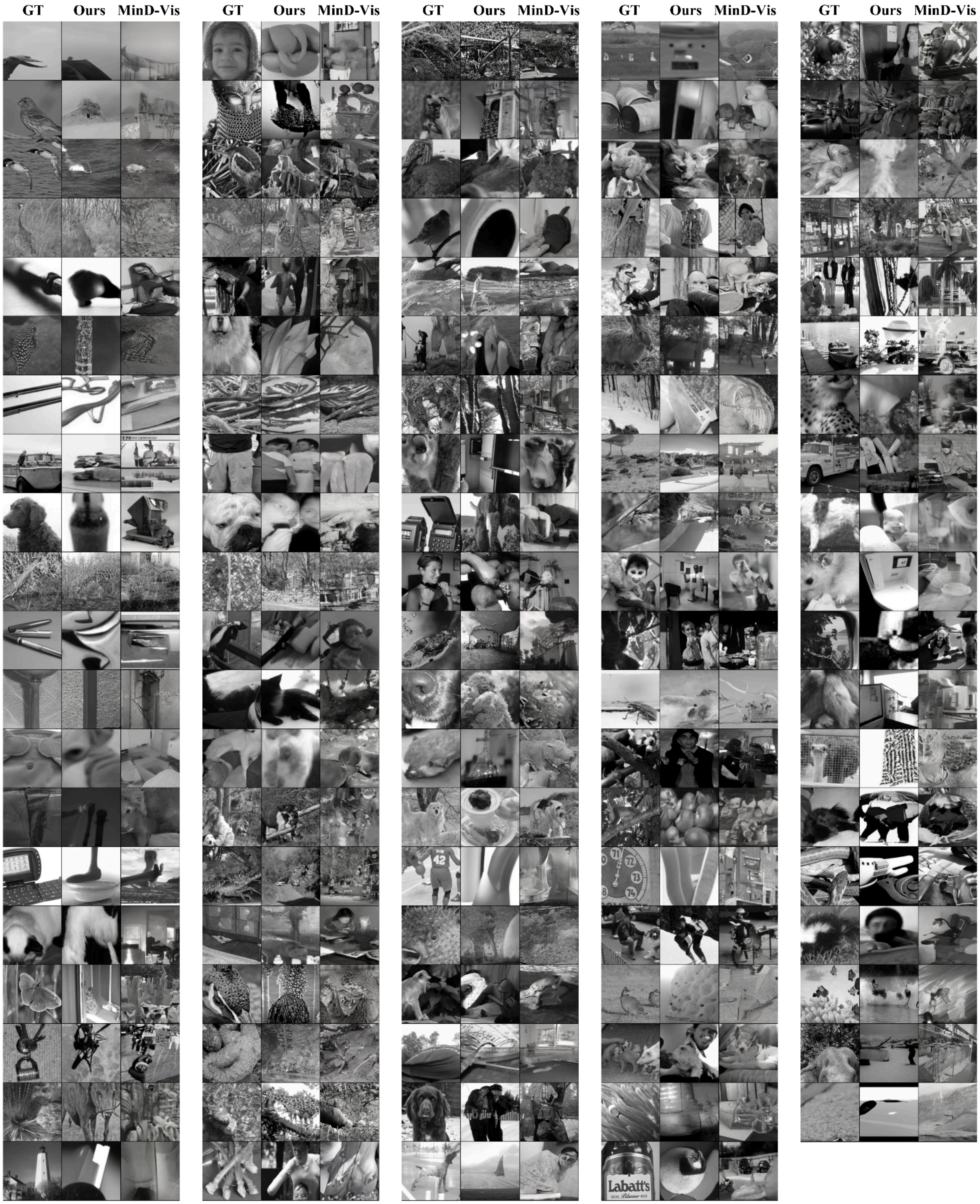

Supplementary Figure 14. The full relationships among the neuron correlations. Similar to Figure 4B, the axis labels indicate which feature (metrics: SSIM, pixel-wise correlation, MSE, LPIPS, and image statistics: brightness, contrast, SI) the neurons are correlated with, and the numbers are the correlation coefficients among these correlation scores.

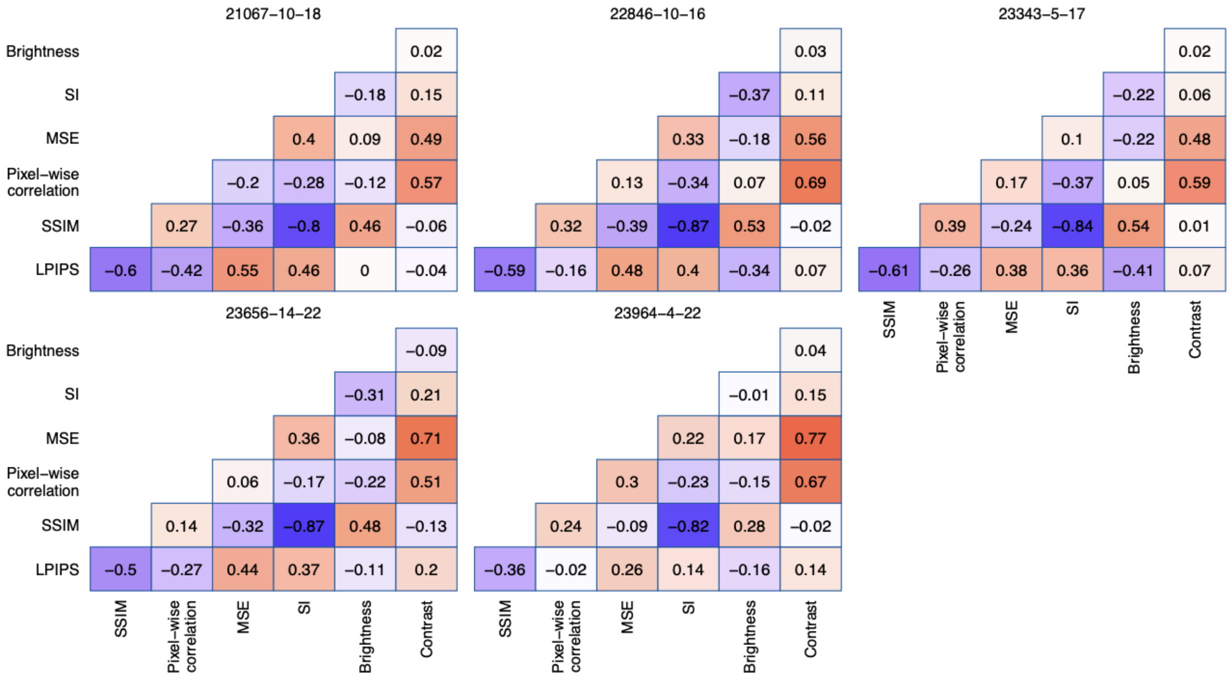

Supplementary Figure 15. The full relationships among the quality metrics, image statistics, and neuron population statistics. The population statistics are calculated from the merged signal values of the neurons responding to the test instances (n = 100). On the x-axis, "IQR" represents the interquartile range; "MAD" represents the mean absolute deviation.

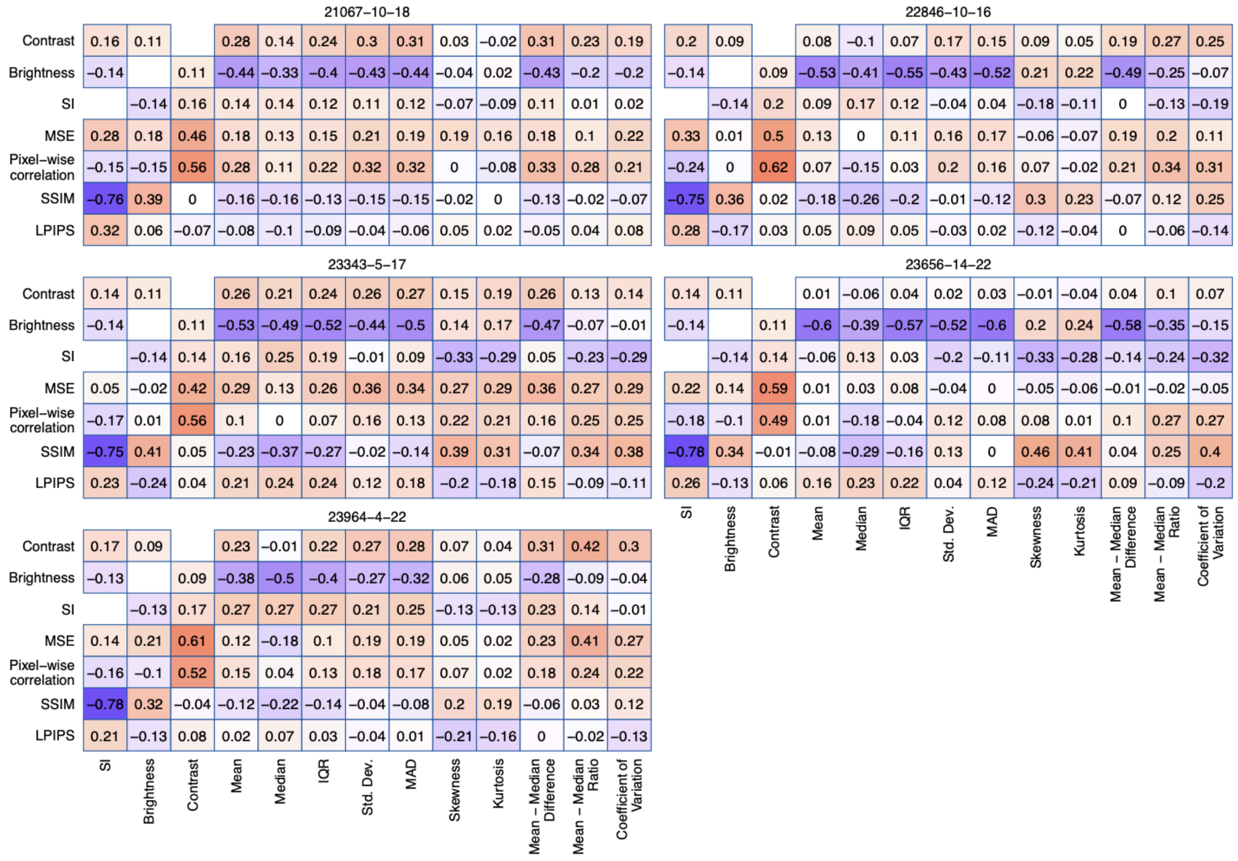

Supplement: Supplementary file 1 — Supporting File: advs74601‐sup‐0001‐SuppMat.pdf [file ADVS-13-e20220-s001.pdf]
